# Design and Experimental Investigation of a 3D Printing Nozzle for Rapid-Hardening Concrete Based on Fluent-EDEM

**DOI:** 10.3390/ma19194072

**Published:** 2026-09-23

**Authors:** Yibo Wang, Sen Xue, Lin Lin, Kun Yang

**Affiliations:** 1 School of Mechanical Engineering and Automation, Liaoning University of Technology, Jinzhou 121000, China; wyxx2157@163.com (S.X.);; 2 School of Civil and Architectural Engineering, Liaoning University of Technology, Jinzhou 121000, China; 3 School of Intelligent Manufacturing, Liaoning Vocational University of Technology, Jinzhou 121000, China

**Keywords:** printing clogging, liquid–solid separation printing, printing nozzle, EDEM discrete element, optimization design, setting, extrusion

## Abstract

To address the clogging issue commonly encountered with rapid-setting and hardening concrete, a liquid–solid separation 3D printing nozzle was proposed based on coupled EDEM–Fluent simulations, and its printing performance was verified. First, finite element models of different nozzle types were established, and Fluent simulations were conducted to analyze the slurry flow velocity and turbulence intensity inside the nozzle, from which the optimal outlet shape was determined. Next, the mixing effectiveness of stirring blades with various structural configurations was evaluated using EDEM, and the optimum combination of blade inclination angle, blade number, and stirring speed was identified through orthogonal experiments. Finally, the liquid–solid separation nozzle was designed, and its printing performance was experimentally validated. The results show that the conical nozzle achieves an outlet velocity standard deviation of 0.0144, indicating favorable velocity uniformity. Using the coefficient of variation as the evaluation index, the lowest value is obtained at a blade inclination angle of 40°, with three blades and a stirring speed of 120 rpm, representing the best mixing performance. In a 1 h continuous printing test, the newly developed nozzle operated without clogging, demonstrating the feasibility of the proposed design for short-duration printing tasks. Moreover, the compressive strength of the printed rapid-setting and hardening specimen (A2) was 14.3% higher than that of the conventionally cast specimen (A1), whereas the flexural strength did not exhibit a significant advantage.

## 1. Introduction

Against the backdrop of the Fourth Industrial Revolution, driven by artificial intelligence, 3D concrete printing technology has emerged as a promising solution to enhance construction efficiency and mitigate the environmental impact of concrete [1]. Conventional extrusion-based deposition processes typically rely on an integrated mixer for batch mixing, requiring the mortar and chemical admixtures to be pre-blended before being fed into the printer hopper. However, the paste formulated with rapid-hardening concrete exhibits an extremely short setting and hardening time [2], making it highly susceptible to premature hardening within the delivery pipeline, which, in turn, leads to persistent clogging issues [3].

To address this challenging issue, extensive research has been conducted by both domestic and international scholars, focusing on areas such as material modification, structural optimization of hopper mixers, and extrusion mechanisms. In terms of material modification, Paul et al. [1] pointed out in their review that the inherent conflict between printability and early-age strength constitutes a core bottleneck. Choong et al. [2] emphasized, from a global perspective, the unique advantages of rapid prototyping technologies in responding to urgent demands. Zhang et al. [3] systematically evaluated the interfacial bonding performance of rapid-hardening, self-compacting concrete and confirmed its significant superiority. Babich et al. [4] investigated the mechanical and deformation properties of high-strength rapid-hardening concrete, revealing the material’s advantages in early-age strength development. With regard to concrete under extreme service conditions, Bolina et al. [5] employed finite element thermal analysis to study the thermal insulation performance of mortars, finding that GFRP- and perlite-based mortars offered the best thermal protection, achieving a nearly 100% temperature reduction, whereas vermiculite–PVC composites performed the worst, with thermal conductivity identified as a critical influencing factor. In the context of mix proportion optimization, Han et al. [6] found that lithium carbonate can significantly shorten the setting time of sulfoaluminate cement and enhance its early-age strength. Regarding extrusion processes and clogging mechanisms, Cheikh et al. [7] identified that maintaining a ratio of the nozzle bottom opening diameter to the maximum aggregate size above 4.25 is critical for preventing aggregate blocking. Cao et al. [8] employed a discrete element model to elucidate the influence of the coarse aggregate volume fraction on pumping pressure and pipe wall wear. Stefan et al. [9] proposed a laboratory-scale paddle mixer and validated the consistency between their discrete element simulation results and experimental data. In terms of nozzle structure design and optimization, Yang et al. [10] reviewed the literature and identified nozzle travel speed, size, shape, and standoff distance from the printing bed as four key parameters affecting printability while also providing operational criteria. They further highlighted clogging and low waste-material recyclability as major sustainability challenges. Currently, conventional screw nozzles, although capable of simultaneous material conveying and mixing, are prone to uneven mixing and extrusion clogging due to the hydration–hardening process and incompressibility of concrete [11]. To tackle this issue, several studies have proposed four types of screw-blade configurations. Through COMSOL simulations and printing experiments, the variable-diameter variable-pitch (VDVP) design was found to enable continuous and uniform extrusion without any clogging throughout the process [12]. In terms of numerical simulation methods, Liu et al. [13] employed EDEM simulations to investigate the mixing performance of a planetary concrete mixer, revealing that although overall mixing uniformity was improved, localized non-uniformity persisted due to unidirectional flow near the sidewalls of the mixing trough. Wang et al. [14] simulated the extrusion flow of natural polymer materials through laminated channels and analyzed the influence of channel geometry on flow behavior. Regarding anti-clogging techniques, Wang et al. [15] identified the primary cause of clogging as the adhesion of partially hardened paste to the screw surface. By applying a superhydrophobic coating to both the screw and nozzle surfaces, they successfully achieved 60 min of continuous, clog-free printing of neat cement paste and reduced the extrusion pressure gradient by 75.07%. However, this coating was found to be susceptible to wear when extruding sand-containing concrete mixes.

Although prior studies have made notable advancements in materials, processing techniques, nozzle design, and numerical simulations, several critical limitations remain unresolved: existing anti-clogging strategies have not addressed the fundamental issue of premature setting within the delivery pipeline; the mixing homogeneity achieved by conventional screw blades is still suboptimal; and the wear resistance of superhydrophobic coatings proves inadequate for sand-containing concrete mixes. To overcome these shortcomings, the liquid–solid separation nozzle proposed herein adopts an active anti-clogging strategy based on in situ mixing and printing, which fundamentally eliminates the risk of pipeline setting. By integrating mixing and extrusion through a differential gearbox, the design effectively shortens the slurry transport path, thereby minimizing residence time and reducing the likelihood of premature hardening. Furthermore, through EDEM–Fluent co-simulation and orthogonal experiments, the optimal blade parameters are determined as an inclination angle of 40°, three blades, and a rotational speed of 120 rpm. Tailored specifically for rapid-hardening cementitious materials, the proposed nozzle achieves a 14.3% increase in compressive strength and enables continuous, clog-free printing for up to one hour. In summary, the developed nozzle demonstrates significant advantages in terms of process integration, optimization methodology, and material compatibility, offering a novel and effective pathway for clogging prevention in 3D printing of rapid-hardening concrete.

## 2. Design of the Liquid–Solid Separation-Type Printing Nozzle

The proposed liquid–solid separation 3D printing nozzle primarily comprises a stepper motor, a differential gearbox, a mixing barrel, solid and liquid feed tubes, stirring blades, an extrusion delivery tube, a screw conveyor, a flow control device, and a nozzle, as illustrated in Figure 1. Its core principle relies on the separate feeding of solid powder and liquid, which are then mixed instantaneously via the differential gearbox and extruded at a constant rotational speed by the screw. Specifically, the solid-phase material is introduced through the powder inlet tube ①, while the liquid-phase material is injected through the liquid inlet tube ② into the mixing barrel. Under high-speed shearing and convective motion generated by the stirring blade assembly ③, the solid and liquid phases are homogenized within 15 s. The resulting paste then passes through the flow control device ④ into the delivery tube ⑤ and is, subsequently, conveyed by the screw to the nozzle for extrusion within approximately 5 s. This total residence time is significantly shorter than the initial setting time of rapid-hardening sulfoaluminate cement paste at ambient temperature, thereby fundamentally eliminating the risk of pipeline clogging at its source.

Considering the specifications of the laboratory platform (1500 mm × 1500 mm × 1600 mm), the structural parameters of the designed printing nozzle are shown in Table 1.

## 3. Simulation Analysis

### 3.1. Nozzle Structural Analysis

As shown in Figure 2, to ensure the material is extruded uniformly and regularly, the Fluent simulation software (version 2022 R2, ANSYS, Inc., Canonsburg, PA, USA) was used to analyze the outlet velocity and turbulence intensity of the material for different nozzle types.

To determine the rheological constitutive equation for subsequent numerical simulations, rheological tests were conducted on the base mix proportion of the concrete. The measured data were transformed and then fitted using four distinct models in Origin (version 2021, OriginLab Corporation, Northampton, MA, USA). Figure 3 presents the relationship between shear rate and shear stress, which serves to evaluate the rheological characteristics of the fresh concrete. Among the four models, the Bingham model, being linear, exhibited the poorest fitting performance. For the other three models, the shear stress increased monotonically with the shear rate; however, once the shear rate exceeded 100 s^−1^, the rate of stress increase gradually diminished, indicating a trend toward stabilization. This behavior is attributed to the thixotropic nature of concrete: as the mixing or shearing rate intensifies, the internal structure of the paste progressively breaks down, leading to a reduced rate of shear stress growth, which eventually approaches a plateau.

The fitting results are summarized in Table 2. The modified Bingham model exhibits a coefficient of determination (R^2^) exceeding 0.996, indicating superior fitting accuracy; it is, therefore, adopted as the constitutive model for the subsequent computational fluid dynamics (CFD) simulations. The rheological equation fitted via Origin is expressed as follows: $\tau =9.91+0.09\gamma -5.41\gamma^{2}$.

To facilitate the simulation within the ANSYS Workbench framework (version 2022 R2), the concrete screw conveying flow is simplified based on the following assumptions: the material is treated as a generalized non-Newtonian, incompressible fluid; the flow is isothermal; gravitational work is taken into account; and the energy equation is neglected. With identical boundary conditions and all other parameters held constant, the influence of different nozzle configurations on the uniformity of the extrusion outlet is systematically investigated. In accordance with the operational process and simulation requirements, a transient analysis is performed. The rotational speed is specified in revolutions per minute (rpm), and the gravitational acceleration is set to −9.8 m/s^2^ in the Y-direction. The material properties—namely, a density of 2400 kg/m^3^ and a viscosity coefficient of 0.09 Pa·s—are derived from experimental measurements and the modified Bingham model fitting, thereby ensuring physical consistency with the actual concrete mix.

From Figure 4, it can be observed that there is no significant overall difference in the flow velocity patterns within the wedge-shaped region among the different nozzle types. However, in the vicinity of the screw, driven by the screw’s rotation, the velocity increases closer to the screw for all four nozzle types, reaching a maximum at the screw tip. This indicates that the screw rotation has a significant influence on the velocity distribution near the nozzles. This phenomenon occurs because the cross-sectional area of the wedge-shaped region awaiting extrusion is considerably larger than the cross-sectional area during screw conveyance. Consequently, the volume of material per unit distance is significantly greater in the wedge region, resulting in smaller displacement distances and lower flow velocities.

As shown in Figure 5, the outlet velocity is lowest at the edge regions for all four nozzle types, while it increases significantly near the center. A comparison of the nozzle outlet velocities reveals that the outlet velocity distribution is relatively uniform for the (a) cylindrical nozzle and the (b) tapered nozzle. In contrast, the outlet velocities for the (c) tapered-diffuser nozzle and the (d) tapered-cylindrical nozzle exhibit a decreasing trend from the center towards the periphery, with distinct velocity gradients. An excessive difference in outlet velocity across the nozzle cross-section is detrimental to achieving uniform material extrusion.

Figure 6 and Figure 7 present the turbulence intensity distribution contours within the flow channels and at the nozzle outlets for the four nozzle types. It can be observed from Figure 6 that, under identical initial conditions, the turbulence intensity distribution patterns for the four different nozzle types are similar to their velocity distribution patterns. Generally, the turbulence intensity is minimal in the wedge-shaped region. As the fluid extrusion approaches closer to the nozzle base, the turbulence intensity increases, with the maximum turbulence intensity concentrated around the screw. This occurs because the cross-sectional area of the nozzle mold continuously decreases, leading to a significant increase in shear interaction among material components, thereby enhancing the turbulence intensity. Furthermore, the fluid material near the screw experiences shear forces from the screw rotation, which increases the irregularity of its motion and further intensifies the fluid’s turbulence.

As shown in Figure 7, the turbulence intensity at the outlet of the Figure 7a cylindrical nozzle exhibits no significant overall variation and is relatively uniform. For the three nozzle types shown in Figure 7b–d, the turbulence intensity at the edge regions is lower than that in the central region. Particularly noteworthy is that, disregarding the variations at the periphery of the nozzle outlet, the turbulence intensity distributions at the outlets of the Figure 7c tapered-diffuser nozzle and the Figure 7d tapered-cylindrical nozzle exhibit a tendency towards “hollowing”, where the turbulence intensity in the center is lower than that in the surrounding areas. This phenomenon is attributed to the wall adhesion effect. The shear interaction between the wall and the fluid material induces a difference in turbulence intensity between the central region and the peripheral region of the fluid.

Using Fluent’s surface integration, the standard deviation of the outlet velocity was calculated to evaluate the uniformity of extrusion. The calculated standard deviations are 0.0079 for the cylindrical nozzle, 0.0144 for the tapered nozzle, 0.0255 for the tapered-diffuser nozzle, and 0.0213 for the tapered-cylindrical nozzle. Since the nozzle geometry influences the flow characteristics of the fluid and, consequently, affects the outlet velocity distribution, the final selection of the nozzle type requires a comprehensive consideration of the outlet flow velocity, turbulence intensity contour distribution, and the standard deviation of the outlet velocity.

Based on a comprehensive evaluation of the simulation results and practical printing requirements, the cylindrical nozzle exhibits a relatively uniform outlet velocity. However, it lacks pressure at the outlet, failing to provide the necessary compaction conditions for the material. This is unfavorable for continuous material extrusion and adversely impacts the quality of the printed specimen. In conclusion, the tapered nozzle is identified as the optimal design solution.

### 3.2. Structural Analysis of Stirring Blades

Concrete is characterized by its heterogeneity and discontinuity, as it is composed of solid particles of various sizes [14]. Investigating the bulk aggregation state among numerous particles is often necessary. The discrete element method (EDEM) can accurately describe features such as inter-particle collisions, cohesive contact, and particle movement trajectories [16]. EDEM enables detailed modeling of the coarse and fine aggregates within concrete, thereby facilitating the simulation of concrete.

Considering the cohesive forces that exist between concrete particles [17], the Hertz–Mindlin model with JKR cohesion was employed to model the concrete particles, using the JKR surface energy to characterize the adhesive effect between particles. Utilizing the Hertz–Mindlin model combined with the JKR model [18] to simulate the mixing process of the concrete mixture allows for a more accurate description of the contact behavior and mechanical properties between particles [19]. The Hertz–Mindlin model accounts for elastic deformation and frictional interactions between particles, while the JKR model describes the adhesive behavior. The calculation of the JKR normal force, Fn, follows Equations (1) and (2) to ensure the accuracy and reliability of the simulation results.

(1) $$F_{n}=-4\sqrt{\pi \gamma E}\alpha^{\frac{3}{2}}+\frac{4E}{3R}\alpha^{3}$$

where *γ* is the surface energy, *E* is the equivalent Young’s modulus, and *R* is the equivalent radius.

The normal overlap, *δ*, and interaction parameters can be calculated as follows:

(2) $$\begin{array}{l} \delta =\frac{\alpha^{2}}{R}-\sqrt{\frac{4\pi \gamma \alpha }{E}}\\ \alpha ={\left[\frac{9\pi R^{2}}{2E}\left(\frac{3}{4}-\frac{1}{\sqrt{2}}\right)\right]}^{\frac{1}{3}} \end{array}$$

The calculation formula for cohesion is the following:

(3) $$F_{n}=-\frac{3}{2}\pi \gamma R$$

Before conducting a simulation analysis using EDEM (version 2022.1, DEM Solutions, Edinburgh, UK), it is necessary to construct a simplified nozzle model, create particles, and design boundary conditions. A three-dimensional model of the concrete 3D printing nozzle was established using SolidWorks (version 2021, Dassault Systèmes, Waltham, MA, USA), divided into .mesh files, and then imported into the Geometry module in EDEM. This provides the foundation for subsequently setting the model’s motion parameters and boundaries. The simplified model is shown in Figure 8.

The primary components of ready-mixed concrete include cement, mineral admixtures, aggregates, water, and various chemical admixtures [20,21], resulting in a relatively complex composition. To simplify the computational and solution processes, the concrete mixing process is approximated by employing two types of spherical particles to simulate the blending of aggregates and cement [22]. The aggregate particle size is set to 3 mm, and the cement particle size to 2 mm, with a total mass ratio of aggregate to cement fixed at 0.8:1. It is noteworthy that the use of spherical particles neglects the mechanical interlocking effects arising from the angularity of real aggregates, which may lead to an overestimation of the absolute mixing efficiency. Therefore, the parametric combination determined in this section should be regarded primarily as a theoretical optimum; practical printing parameters require fine-tuning in conjunction with the actual forming quality observed in the printing experiments presented in Section 4. The material properties of the two aggregates are presented in Table 3.

In the Geometry panel of the pre-processor, import the completed mixing zone model, set the mixing blades to “Add Linear Rotation Kinematic”, create a particle factory, and define the virtual particle generation position, generation velocity, and particle mass. The spherical particle size settings in EDEM are shown in Figure 9 (only the 2 mm particle size is listed here as an example).

According to the literature [23], the angle of repose of the sand–gravel mixture was measured using the injection method. Based on the scale of the concrete 3D printing nozzle and the angle of repose of the blended constituent materials, the corresponding parameters were input into the GEMM (Generic EDEM Material Model) database. By further consulting the GEMM general database, as reported in References [24,25,26], the fundamental contact parameters and JKR surface energy parameters for the discrete element modeling of concrete particles were ultimately determined, as shown in Table 4.

The charging sequence involves adding the aggregate first, followed by the cement. The Rayleigh time step is set to 22%, and the mixing simulation time is configured for 20 s. Different colors are used to define distinct particle types (red for aggregate and blue for cement) to visually track the distribution state of the two particle types during the mixing process.

Concrete is a cementitious material [27], characterized by a high inter-particle viscosity coefficient. As the mixer continuously rotates the mixture, the viscous forces within the concrete mix and the friction between particles constitute the primary resistance to the mixing process [28]. Different mixer blade types exhibit significantly different axial and radial force effects. Figure 10 illustrates three widely used blade types.

Based on the material parameters of concrete particles and the simulation boundary conditions, the mixing performances of different blade types were simulated. Experiments with a total of three simulation groups were conducted, with each experimental time period set from T = 0 to 20 s. The simulation parameters were fixed as single variables; specifically, each blade type was configured with three blades, a blade inclination angle of 45°, and a rotational speed of 100 rpm to investigate the changes in particle distribution during the mixing process. Simulation analyses were performed under identical variable conditions, except for the blade type. Figure 11, Figure 12 and Figure 13 show the simulation processes for the frame-type, paddle-type, and blade-type mixers, respectively.

Figure 11 illustrates the simulation process for particle mixing using frame-type blades at various times. Taking T = 0 s as an example, the location of the cement particles (blue particles) is referred to as the upper mixing zone, while the aggregate particles (red particles) occupy the lower mixing zone. At T = 0 s, with frame-type blade mixing, the aggregate and cement particles have just been charged, and mixing has not yet commenced. The two particle types are distinctly stratified, with only slight contact occurring at the interface. During the period from T = 0 s to T = 4 s, as the simulation progresses, the aggregate particles slowly diffuse upward from the bottom of the lower mixing zone after charging. The particle motion is gentle, and the aggregate particles scarcely reach the upper mixing zone. At T = 20 s, an inefficient mixing zone is clearly evident in the lower region, indicating that the mixing performance is not ideal.

Figure 12 illustrates the simulation process for particle mixing using paddle-type blades at various times. Compared to the frame-type blades, during the paddle-type mixing process, the range of particle diffusion toward the top increases, and the presence of aggregate particles can be clearly observed in the upper mixing zone. At a simulation time of T = 20 s, the density of the inefficient mixing zone at the bottom is reduced compared to that of the frame-type blades; however, localized aggregation of aggregate particles still persists.

Figure 13 depicts the simulation process for particle mixing using fan-type blades at various times. By T = 4 s, the mixing effect of the materials within the mixing zone has become significantly more pronounced compared to the first 4 s; the stratification phenomenon has disappeared, and the axial movement of particles is vigorous. During the period from T = 8 s to T = 20 s, the mixing intensity gradually stabilizes, and the range of particle motion within the mixing zone is broader than that observed with the previous two blade types, thereby achieving the optimal mixing performance.

To further elucidate the mixing performance of the three different blade types, randomly selected particles with the same identifier were analyzed in terms of their motion trajectory and pressure distribution at a mixing time of T = 20 s, as shown in Figure 14 and Figure 15.

It can be observed from Figure 14c that the trajectory of the selected particle within the fan-type blade mixing zone is complex. In the top view direction, the particle undergoes a large-scale circular motion within the mixing zone while simultaneously exhibiting small-scale circular movements. As shown in Figure 14a,b, the trajectories of particles in the other two blade types are merely characterized by regular vertical movement around the central axis. Examination of the front view reveals that within the fan-type blade mixing zone, the particle not only moves in a circular path but also experiences relatively vigorous axial motion.

As shown in Figure 15, the particles are compressed to a certain extent at the bottom of the mixing zone due to the extrusion effect of the fan-type mixing blades. Overall, the motion trajectory of the particles within the mixing zone is relatively ideal, essentially filling the entire mixing space.

The coefficient of variation (CV) is adopted as the quantitative index for evaluating mixing uniformity, with a lower CV indicating superior mixing performance. Concurrently, range analysis is employed to assess the influence degree of each factor, where a larger range corresponds to a more pronounced effect. During the simulation, the mixing zone is discretized into a grid system, and the numbers of aggregate particles, cement particles, and total particles within each grid are recorded at one-second intervals. After excluding invalid grids with a total particle count of less than 20, the CV values are computed for various parameter combinations, revealing significant differences among the tested conditions.

Assume that the total numbers of aggregate and cement particles across all grids are denoted as G and S, respectively, and the overall total particle count is Z=G+S. At a given instant, let g and s represent the numbers of aggregate and cement particles within a specific grid, and, thus, the total particle count in that grid is z=g+s. The aggregate proportion in each grid, denoted as a, is defined as follows:

(4) $$a=\frac{g}{z}$$

The optimal aggregate proportion of A under ideal conditions is given by the following:

(5) $$A=\frac{G}{Z}$$

The deviation of the aggregate within a single grid from an ideally uniform mixture is defined as follows:

(6) $$\varepsilon_{i}=\frac{a_{i}}{z}$$

The standard deviation of the aggregate is calculated as follows:

(7) $$S_{X}=\sqrt{\frac{\Sigma_{i=1}^{n}{\left(\varepsilon_{i}-\bar{\varepsilon }\right)}^{2}}{n-1}}$$

(8) $$\bar{\varepsilon }=\frac{\sum_{i=1}^{n}\varepsilon_{i}}{n}$$

where n = 1, 2, 3, …

The coefficient of variation in the aggregate, $C_{v}$, is then expressed as follows:

(9) $$C_{v}=\frac{S_{X}}{\bar{\varepsilon }}$$

Similarly, the coefficient of variation in the cement particles at a given moment can be obtained.

Based on the simulation results for the three blade types, the corresponding coefficients of variation were output, as shown in Figure 16. The coefficient of variation for the fan-type mixing blades exhibits the most dramatic decrease over time. At T = 10 s, its coefficient of variation is significantly lower than those of the paddle-type and frame-type blades, indicating that the fan-type achieves the best mixing performance. In contrast, the coefficient of variation values for the paddle-type and frame-type blades are comparable, suggesting little difference in their mixing effectiveness. Through a comprehensive analysis of particle motion trajectories, compressive forces experienced, and the coefficient of variation, it is concluded that the fan-type blade represents the optimal mixing blade configuration.

### 3.3. Optimization of Structural Parameters

To design a liquid–solid separation 3D printing nozzle suitable for rapid-setting and rapid-hardening concrete materials, it is necessary to further determine the range of values for the blade structural parameters and mixing parameters while meeting the design requirements of the mixing blades. Therefore, an orthogonal experimental method was adopted to establish the parameter ranges [29], as shown in Table 5.

The value ranges for the blade inclination angle, number of blades, and stirring speed were determined based on the following considerations: (1) the typical parameter ranges reported in Reference [30] and similar concrete mixing studies; (2) the constraint imposed by the structural dimensions of the present nozzle (stirring zone diameter of 150 mm) on the stirring speed; and (3) a preliminary exploration of the effective parameter ranges through pre-experiments. Based on the above, the blade inclination angle was set in the range of 35–45°, the number of blades was set between three and five, and the stirring speed was set between 100 and 140 r/min, ensuring that the selected ranges cover representative levels within the feasible engineering scope.

After determining the experimental factors, an L9(33) orthogonal array was formulated based on the orthogonal experimental design table to guide the implementation of the experiments. The blade and mixing zone models required for the nine test groups were established accordingly, as shown in Table 6.

For each set of experiments, the particle velocity vector diagrams of the concrete mixing process were captured at T = 10 s and T = 20 s for analysis, as shown in Figure 17 and Figure 18.

As illustrated in Figure 17 and Figure 18, driven by the rotation of the mixing blades, the aggregate and cement particles undergo continuous motion and mixing. The cement particles uniformly coat the aggregate, resulting in a generally satisfactory mixing performance. Upon closer observation, it can be noted that a slight accumulation of aggregate occurred at the bottom of the mixing zone in Test Group 2 and in the middle of the mixing zone in Test Group 9, indicating that the mixing performance in these groups was inferior compared with the others. In contrast, Test Groups 4, 7, and 8 exhibited a more uniform dispersion of aggregate and better overall mixing performance relative to the other groups.

The data from each group were calculated to obtain the coefficient of variation. The letters A, B, and C are used to represent the blade inclination angle, number of blades, and mixing speed, respectively, as shown in Table 7. The trend in the coefficient of variation for each test group is illustrated in Figure 19.

The range analysis presented in Table 8 reveals that the degree of influence of the factors on the result follows the order: B > A > C. Accordingly, the combination that yields the smallest coefficient of variation is B1A2C2, which corresponds to Trial 4 in Table 7, with a minimum coefficient of variation of 0.2294.

Based on a comprehensive analysis of Table 6 and Figure 19, it can be concluded that the lowest coefficient of variation and, thus, the optimal mixing performance was achieved under the parameter conditions of Test Group 4. Therefore, the optimal fan-type blade structural parameters and mixing parameters are determined to be a blade inclination angle of 40°, three blades, and a mixing speed of 120 rpm.

## 4. Printing Performance Test

To verify the printing performance of the liquid–solid separation 3D printing nozzle, a prototype was designed and fabricated as shown in Figure 20. It mainly consists of several components, including a stepper motor, a gearbox, a filament feeding mechanism, a mixing device, a flow control device, and a feeding device.

### 4.1. Material Preparation and Specimen Fabrication

The rapid-setting and rapid-hardening concrete (3DP) material is primarily formulated with specific proportions of rapid-setting and rapid-hardening sulfoaluminate cement (SAC, Grade 42.5, Yangchun Company, China), aggregate (ordinary river sand from Bohai Cement Building Materials Co., Ltd., Jinzhou, China), mineral powder (MP, Longze Company, China), fly ash (FA, Longze Company, China), Hydroxypropyl methylcellulose (HPMC, ShangKang Chemical, China), water reducer WR (Huake, Guangzhou, China), and lithium carbonate (Qiantelu Chemical, Zhengzhou, China) as an accelerator, among other components. The physical and mechanical performance indicators for the SAC cement material are shown in Table 9.

The base mix proportion of the printing paste is sulfoaluminate cement:mineral powder:fly ash = 1:0.41:0.41, with the dosage of admixtures calculated as a percentage of the cementitious materials by mass, as detailed in Table 10.

Based on the proportion of Li_2_CO_3_ added to the concrete material, it is classified into ordinary concrete and rapid-setting and rapid-hardening concrete. The rapid-setting and rapid-hardening concrete material with a Li_2_CO_3_ ratio of 0.225% was selected for the fabrication of printed specimens using the liquid–solid separation printing process and for traditionally cast specimens.

The length of the printed specimen was set to 160 mm, the height to 48 mm, and the printing layer height to 12 mm. The screw rotation speed was determined to be 60 rpm, the nozzle diameter was 25 mm, and the printing speed range was set at 20 mm/s. Group A1 (Li_2_CO_3_ at 0%) was designated as the printed specimen for ordinary concrete, while Group A2 (Li_2_CO_3_ at 0.225%) was designated as the specimen for rapid-setting and rapid-hardening concrete. To ensure the scientific validity of the comparative test, the mix proportion for the cast specimen was kept consistent with that of the rapid-setting and rapid-hardening type; therefore, its group was set as A2-1 (Li_2_CO_3_ at 0.225%).

To match the fixed screw speed of 60 rpm, printing travel speeds of 5 mm/s, 10 mm/s, 15 mm/s, and 20 mm/s were used for debugging. The A2 group material was selected for this purpose. The presence or absence of obvious grooves and cracks on the surface of the printed specimen was used as the criterion for evaluating the matching effect of the printing speed. The printing effects are shown in Figure 21. As can be seen from Figure 21, at different printing speeds, the liquid–solid separation 3D printing nozzle enabled smooth and efficient printing of the rapid-setting and rapid-hardening concrete material, without any occurrence of clogging issues.

After curing the printed specimens for 7 days, the surface quality of the specimens was obtained, as shown in Figure 22. It can be observed that at speeds of 5 mm/s and 10 mm/s, the surface formation quality is smooth and even, without obvious cracks or irregular surface patterns. However, at a printing speed of 5 mm/s, due to the excessively slow printing speed, the volume of paste extruded by the screw exceeded the actual required amount, making it easy for the concrete to spread to the surroundings, thereby increasing the width of the printed specimen (Figure 22a). Therefore, the printing speed was set to 10 mm/s.

The specimen dimensions were set to 40 mm × 40 mm × 40 mm for the compressive strength tests. For the flexural strength tests, the specimen dimensions were set to 40 mm × 40 mm × 160 mm, as shown in Figure 23. Internationally standard specimen molds were used to prepare the cast concrete specimens [31], as illustrated in Figure 24. All specimens were cured for 7 days and 28 days. To ensure the statistical reliability of the test data, three compressive specimens and three flexural specimens were prepared for each group. All specimens were cured under standard curing conditions (temperature of 20 ± 2 °C, relative humidity ≥ 95%) until the specified age. The test results are expressed as the arithmetic mean ± standard deviation.

The test was conducted in accordance with the national standard GB/T 17671-2021, “Test Method for Strength of Cement Mortar (ISO Method)” [32], using a CMT5305 electronic universal testing machine manufactured by MTS Industrial Systems (China) Co., Ltd., for the mechanical strength tests.

### 4.2. Results and Discussion

To verify the long-term anti-clogging performance of the liquid–solid separation nozzle, a 1 h continuous printing test was designed and conducted. The test conditions were as follows: screw speed of 60 rpm, stirring speed of 120 rpm, nozzle diameter of 25 mm, and printing speed of 10 mm/s. During the test, the extrusion pressure, screw torque, and extrusion mass flow rate were recorded at 10 min intervals to monitor the stability of the operational performance. The clogging criteria were defined as follows: (1) extrusion interruption or a sudden drop in the extruded amount exceeding 20% of the initial value; (2) an abnormal increase in the screw torque exceeding 30% of the initial value; and (3) the appearance of obvious discontinuities or tensile cracks in the printed filament. As shown in Table 11, during the 60 min of continuous operation, the extrusion pressure remained stable within the range of 0.52–0.61 MPa.

As shown in Table 11, during the 60 min of continuous operation, the extrusion pressure remained stable within the range of 0.52–0.61 MPa, the screw torque increased slightly from 2.4 to 3.0 N·m (within the allowable fluctuation range), and the extrusion mass flow rate decreased from 28.6 to 27.0 g/s, still meeting the printing requirements. The extruded filament width remained stable at around 25.0–25.6 mm, and no clogging occurred throughout the test. The above data provide quantitative support for the anti-clogging performance of the nozzle.

Figure 25 shows the compressive loading test process for the three groups of concrete specimens at the same age during the compressive strength test. During the compressive test, the loading platen of the testing machine applies the load downward at a uniform speed. The resulting stress induces linear elastic deformation in the specimen, and the concrete specimen begins to exhibit significant lateral expansion. As the loading platen continues to descend, the specimen enters a non-linear deformation stage, gradually losing elasticity and beginning to undergo plastic deformation, accompanied by debris spalling from the surface around the concrete specimen. When the load reaches the ultimate load-bearing capacity of the specimen, failure occurs.

Figure 26 presents a comparison of the compressive strength performances of the three groups of concrete specimens of the same age. As shown in Figure 25, the 7-day and 28-day compressive strengths of the ordinary concrete 3D-printed specimens in Group A1 are 8.4 MPa and 13.1 MPa, respectively; the 7-day and 28-day compressive strengths of the rapid-setting and rapid-hardening concrete 3D-printed specimens in Group A2 are 9.6 MPa and 12.1 MPa, respectively; and the 7-day and 28-day compressive strengths of the cast specimens made with the same rapid-setting and rapid-hardening concrete mix in Group A2-1 are 9.3 MPa and 13.2 MPa, respectively.

The experimental results indicate that the 7-day strength of the rapid-setting and rapid-hardening concrete 3D-printed specimens (Group A2) increased by 14.3% compared with the ordinary concrete printed specimens (Group A1). Furthermore, its early strength already reached approximately 79.3% of its 28-day strength value, demonstrating significant early strength characteristics. Although the 28-day strength of Group A2 is 7.6% lower than that of Group A1, its strength-retention rate reaches 91.7% compared with the cast specimens with the same mix proportions (Group A2-1). This indicates that the rapid-setting and rapid-hardening material can maintain good strength performance during the 3D printing process. Through this comparison, the experiment highlights the early strength advantage of rapid-setting and rapid-hardening concrete materials in the context of 3D printing.

Figure 27 illustrates the flexural loading test process for the three groups of concrete specimens of the same age during the flexural strength test. The specimen deforms under the gradually increasing bending load, with the degree of deformation being proportional to the magnitude of the load. As the loading platen of the testing machine continues to descend, the stress at the bottom of the specimen gradually exceeds the tensile strength of the concrete, leading to crack initiation in the specimen. These cracks originate at the bottom of the specimen and progressively propagate upwards until the specimen fails.

Figure 28 presents a comparison of the flexural strength performances of the three groups of concrete specimens of the same age. As can be seen from Figure 27, the 7-day and 14-day flexural strengths of the ordinary concrete 3D-printed specimens in Group A1 are 4.6 MPa and 7.4 MPa, respectively; the 7-day and 14-day flexural strengths of the rapid-setting and rapid-hardening concrete 3D-printed specimens in Group A2 are 5.3 MPa and 6.9 MPa, respectively; and the 7-day and 14-day flexural strengths of the cast specimens in Group A2-1 are 4.8 MPa and 7.2 MPa, respectively.

Although the rapid-setting and rapid-hardening concrete 3D-printed specimens did not demonstrate a significant advantage in flexural strength, the experimental results still hold important research value. Firstly, the rapid-setting and rapid-hardening concrete exhibits favorable early-age strength characteristics in terms of compressive strength, suggesting its potential for more efficient construction in practical engineering applications. Secondly, by comparing and analyzing the differences in mechanical properties under different processing conditions, a scientific basis can be provided for optimizing the formulation of 3D-printed concrete and the construction process. Despite the lack of a significant improvement in flexural strength, the test results also indicate that the addition of Li_2_CO_3_ can enhance the early-age flexural performance of concrete specimens to a certain extent. Furthermore, comparing Groups A2 and A2-1 reveals that the 28-day strength of the 3D-printed specimens is slightly higher than that of the cast specimens.

A mechanism analysis indicates that the significant enhancement in compressive strength of the 3D printed concrete by the novel liquid–solid separation nozzle primarily stems from improved slurry mixing uniformity and more efficient interfacial compressive stress transfer between layers. In contrast, the limited improvement in flexural strength is attributed to the inherent weakness of interlayer interfaces, the anisotropy of mechanical properties, and the high brittleness of the rapid-hardening material. These factors collectively hinder the effective suppression of crack initiation and propagation in the tensile zone at the bottom of specimens under bending loads.

## 5. Conclusions

This paper proposes a novel liquid–solid separation 3D printing nozzle structure, optimizes its structural parameters, and, ultimately, validates the practical performance of the nozzle through printing experiments. The following conclusions are drawn:

(1) Within a typical printing duration of one hour, the nozzle significantly reduces the clogging risk of rapid-hardening concrete slurry and enhances the early mechanical strength of printed specimens. Experimental and simulation analyses further reveal the flow and mixing characteristics of rapid-setting and -hardening concrete slurry during the liquid–solid separation 3D printing process.
(2) Combining finite element (simulating fluid flow) and discrete element (simulating particle motion) methods, the influence of different mixing blade types and structural parameters on particle motion trajectories and mixing performance was analyzed. Through orthogonal experiments, it was determined that the optimal mixing performance is achieved with a blade inclination angle of 40°, three blades, and a mixing speed of 120 rpm.
(3) Under the liquid–solid separation printing process, the rapid-hardening concrete specimens exhibited a 14.3% higher 7-day compressive strength (9.6 MPa) compared with the ordinary concrete specimens (8.4 MPa), indicating that the rapid-hardening material is well-suited for this printing process. The 28-day compressive strength of the printed rapid-hardening specimens reached 91.7% of that of the cast specimens with the same mix proportions, demonstrating good strength retention. However, it should be noted that the present study did not include a direct control experiment using a conventional integrally mixed 3D printing nozzle under identical material conditions. Therefore, the performance comparison between the proposed nozzle and conventional printing nozzles requires further validation through controlled experimental studies

## Figures and Tables

**Figure 1:**
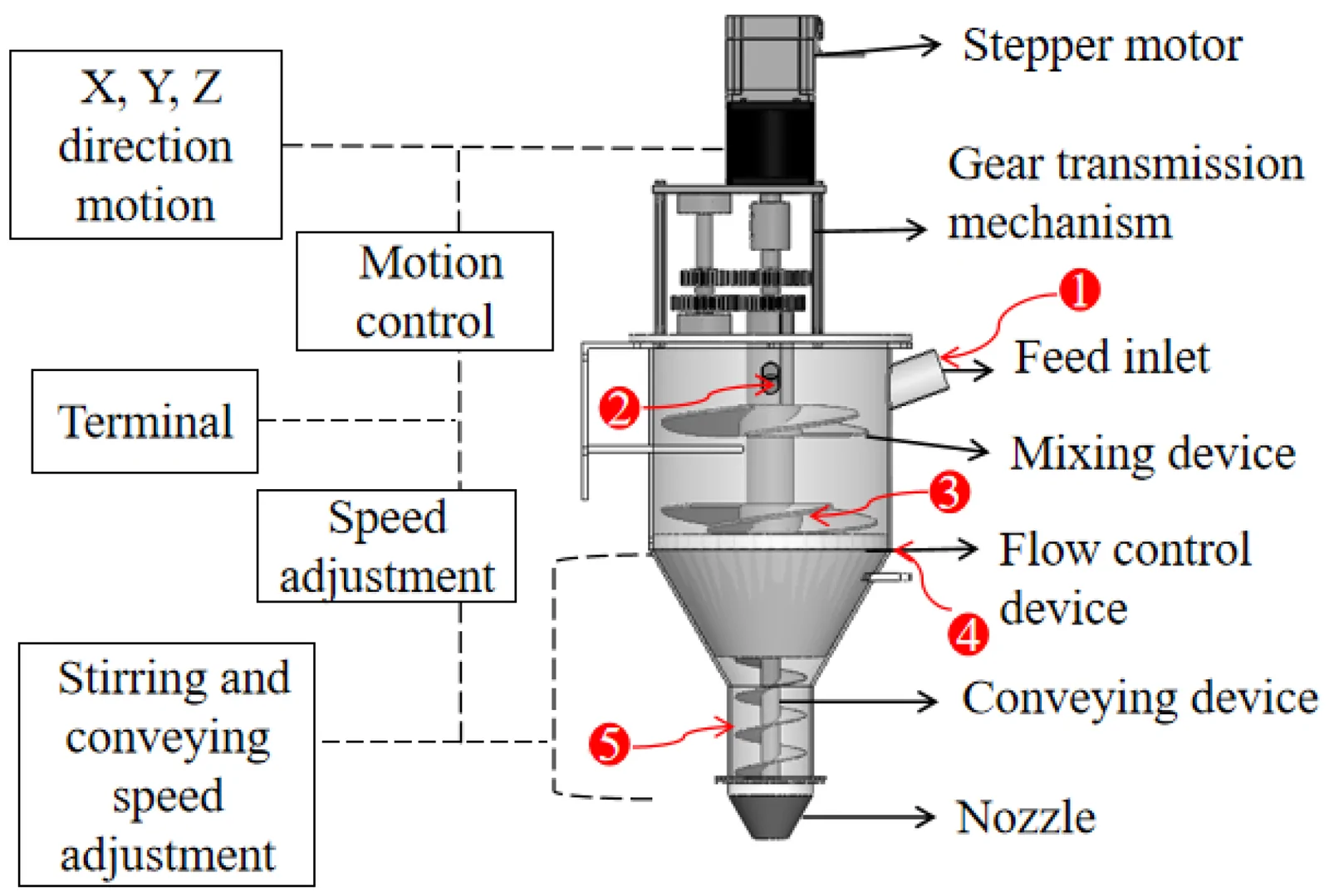
Structural diagram of the liquid–solid separation printing nozzle.

**Figure 2:**
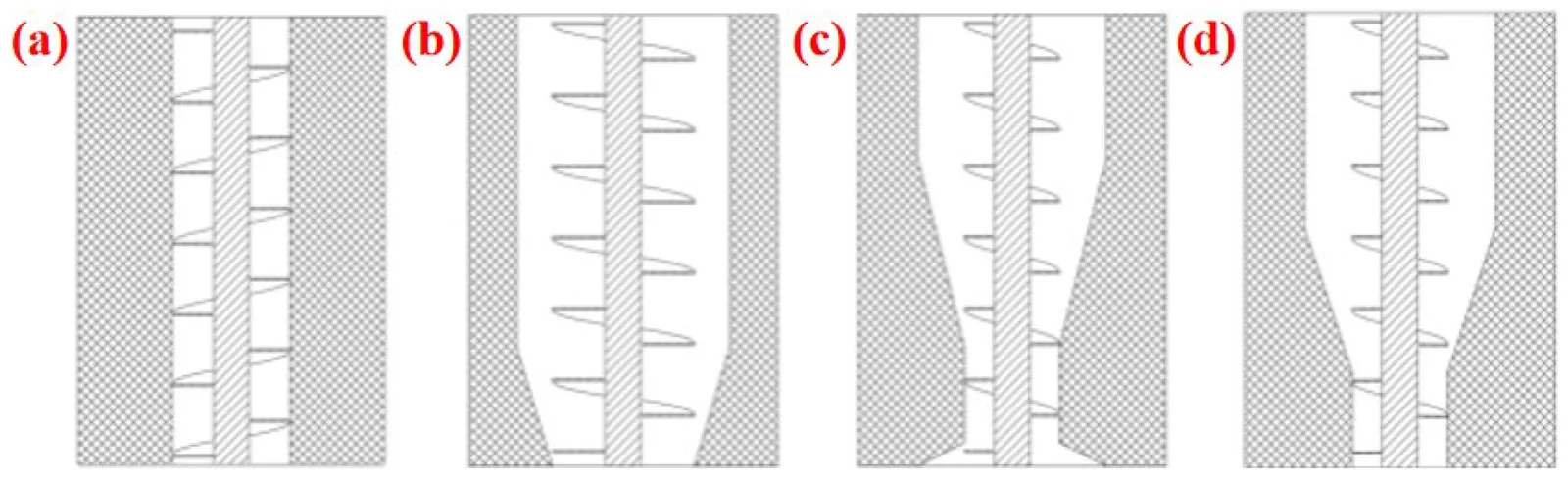
Nozzle structural diagram: (**a**) cylindrical type; (**b**) cone type; (**c**) cone cylinder diffusion type; (**d**) cone cylinder type.

**Figure 3:**
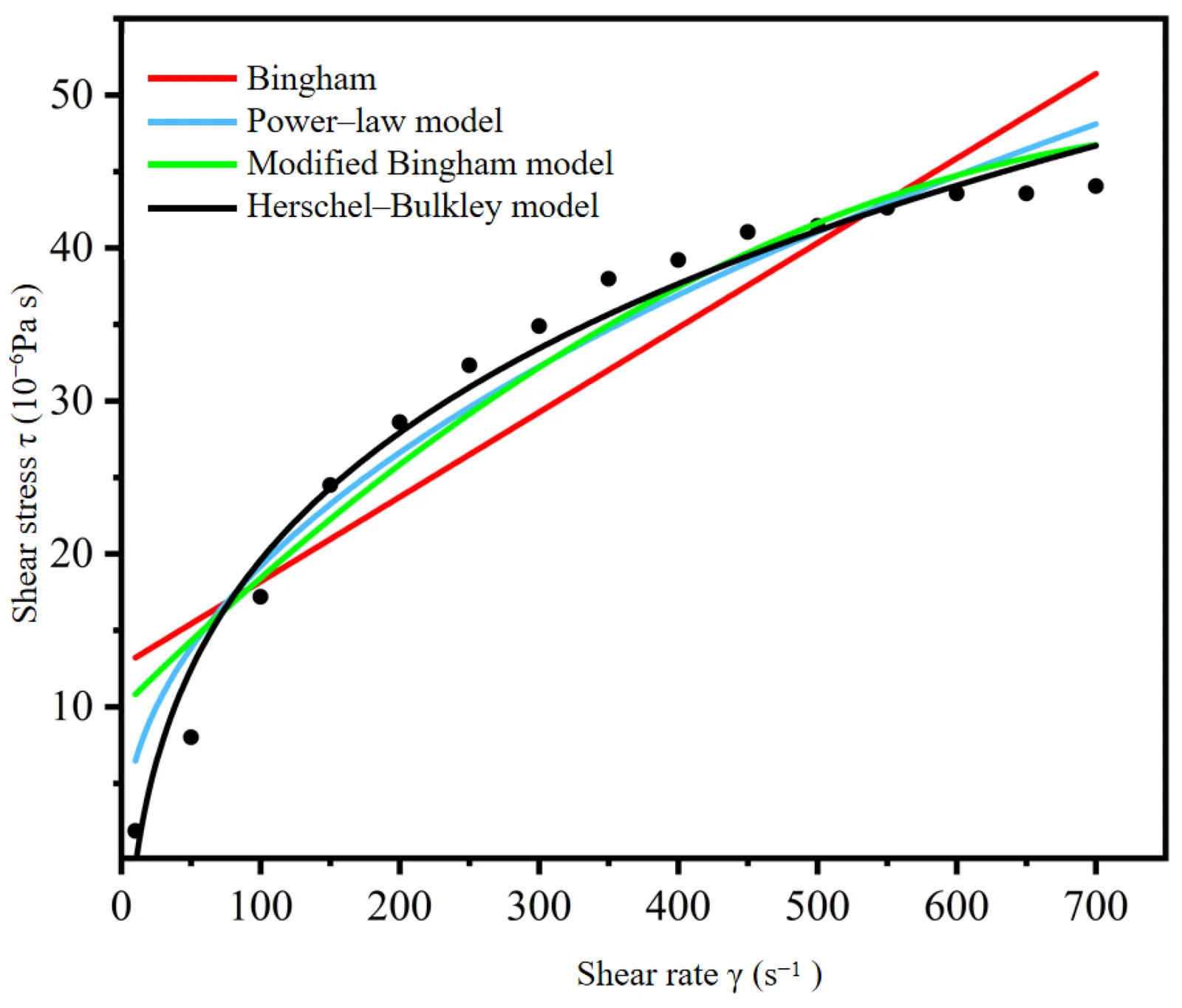
Data model fitting.

**Figure 4:**
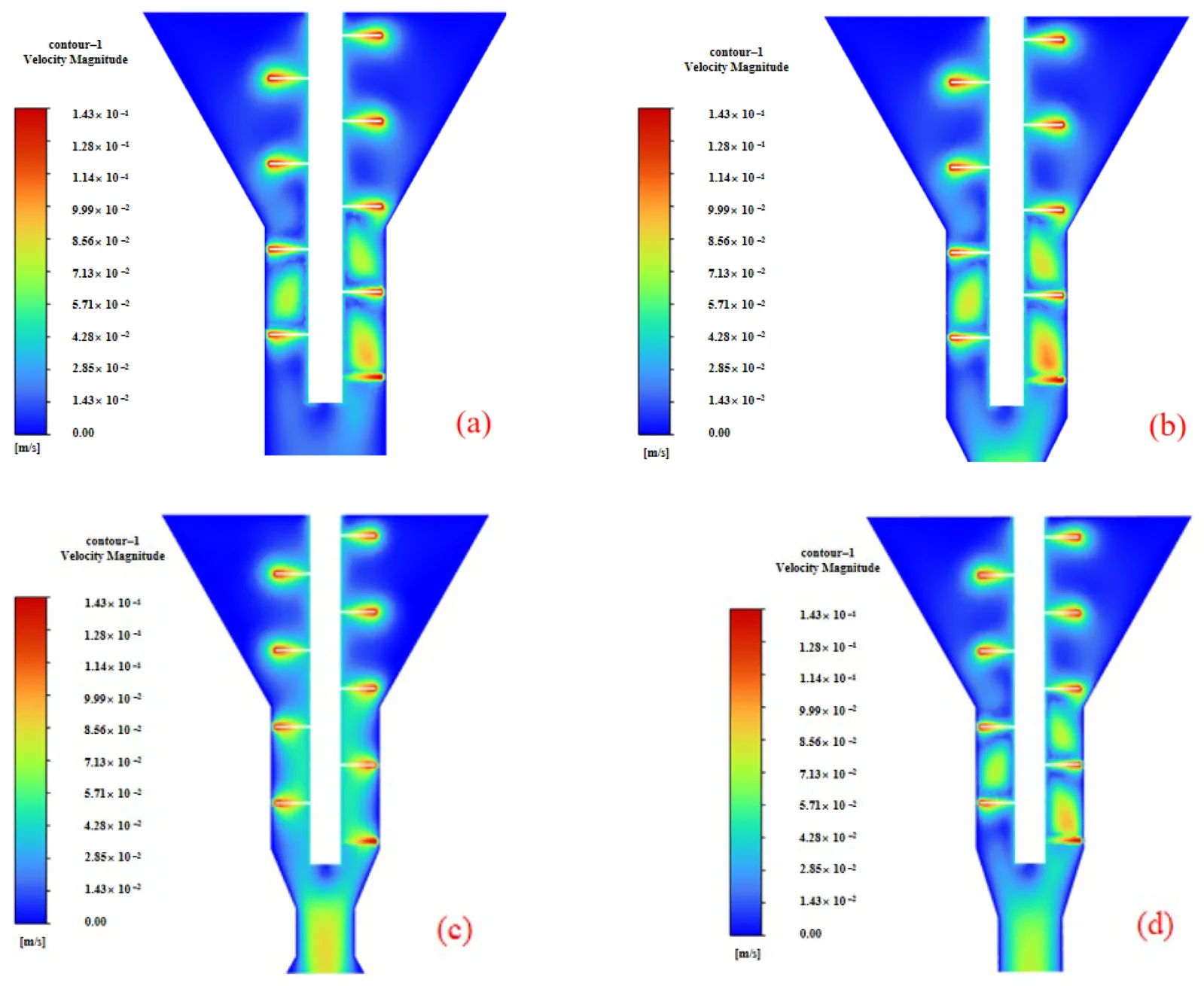
Cloud map of the velocity distribution in the four nozzle channels. (**a**) Cylindrical type (**b**) Tapered type (**c**) Tapered-diffuser type (**d**) Tapered-cylindrical type.

**Figure 5:**
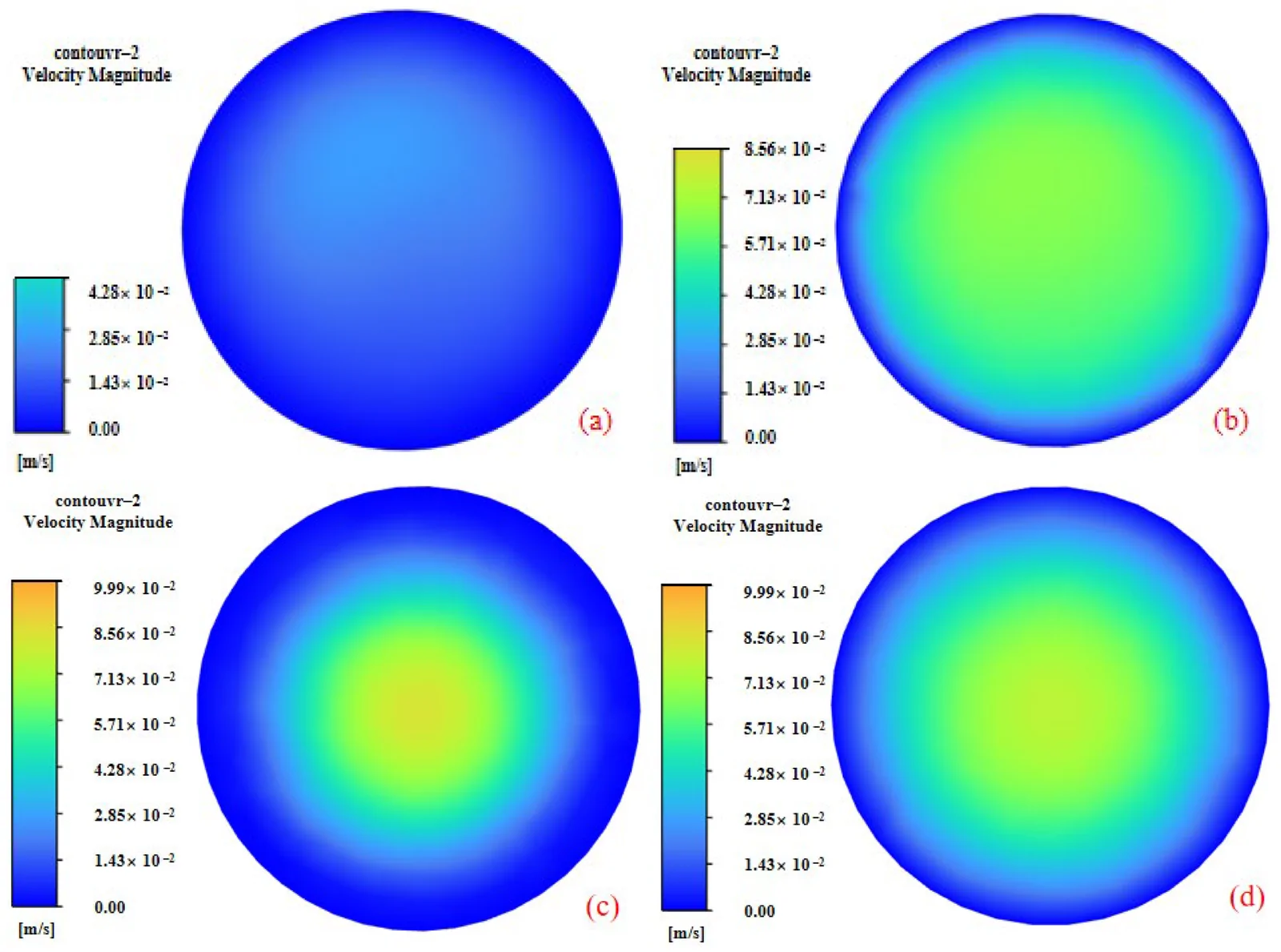
Cloud maps of the velocity distribution at the outlets of the four nozzle channels. (**a**) Cylindrical type (**b**) Tapered type (**c**) Tapered-diffuser type (**d**) Tapered-cylindrical type.

**Figure 6:**
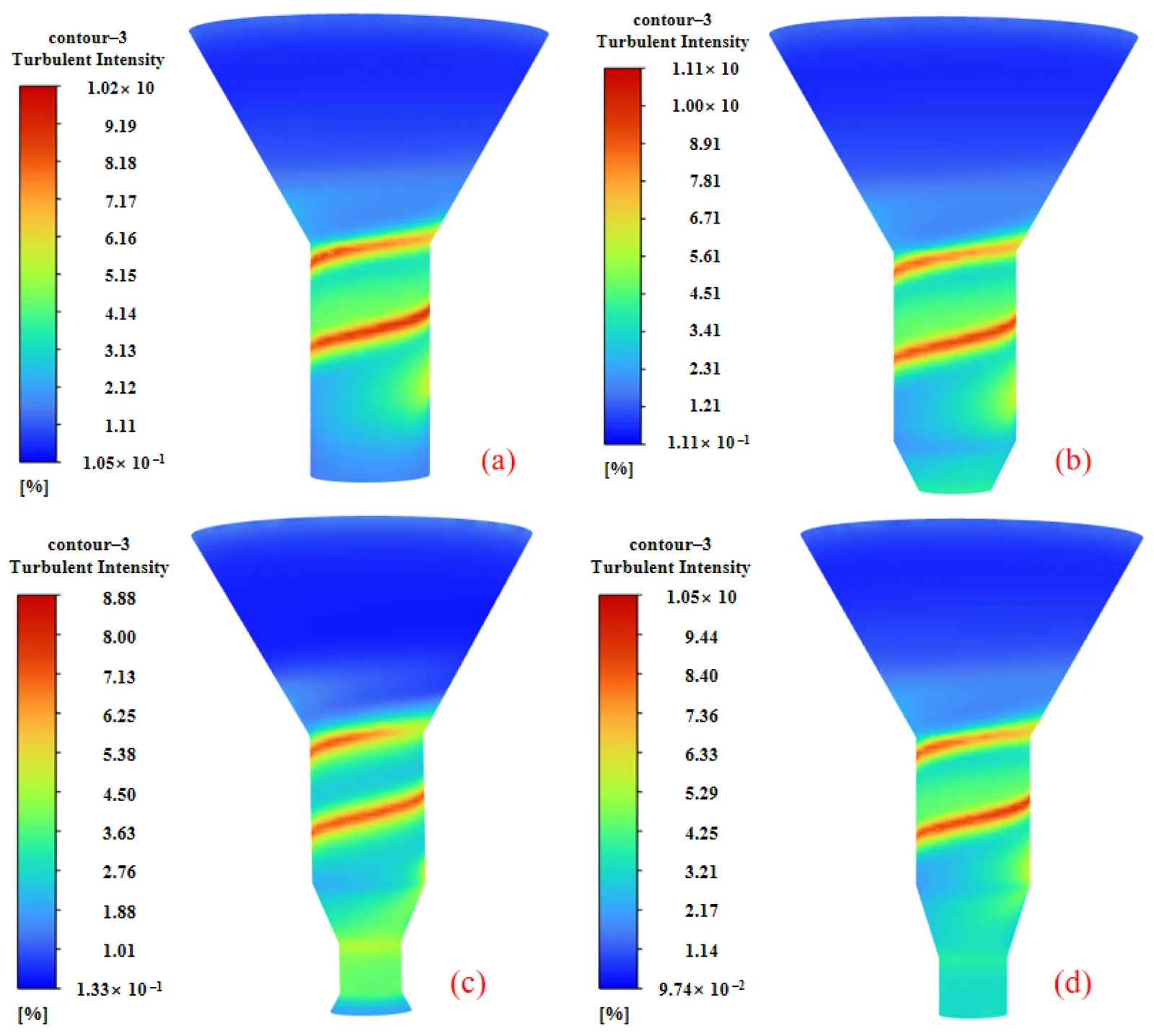
Cloud maps of the turbulence intensity distribution on the walls of the four types of nozzles. (**a**) Cylindrical type (**b**) Tapered type (**c**) Tapered-diffuser type (**d**) Tapered-cylindrical type.

**Figure 7:**
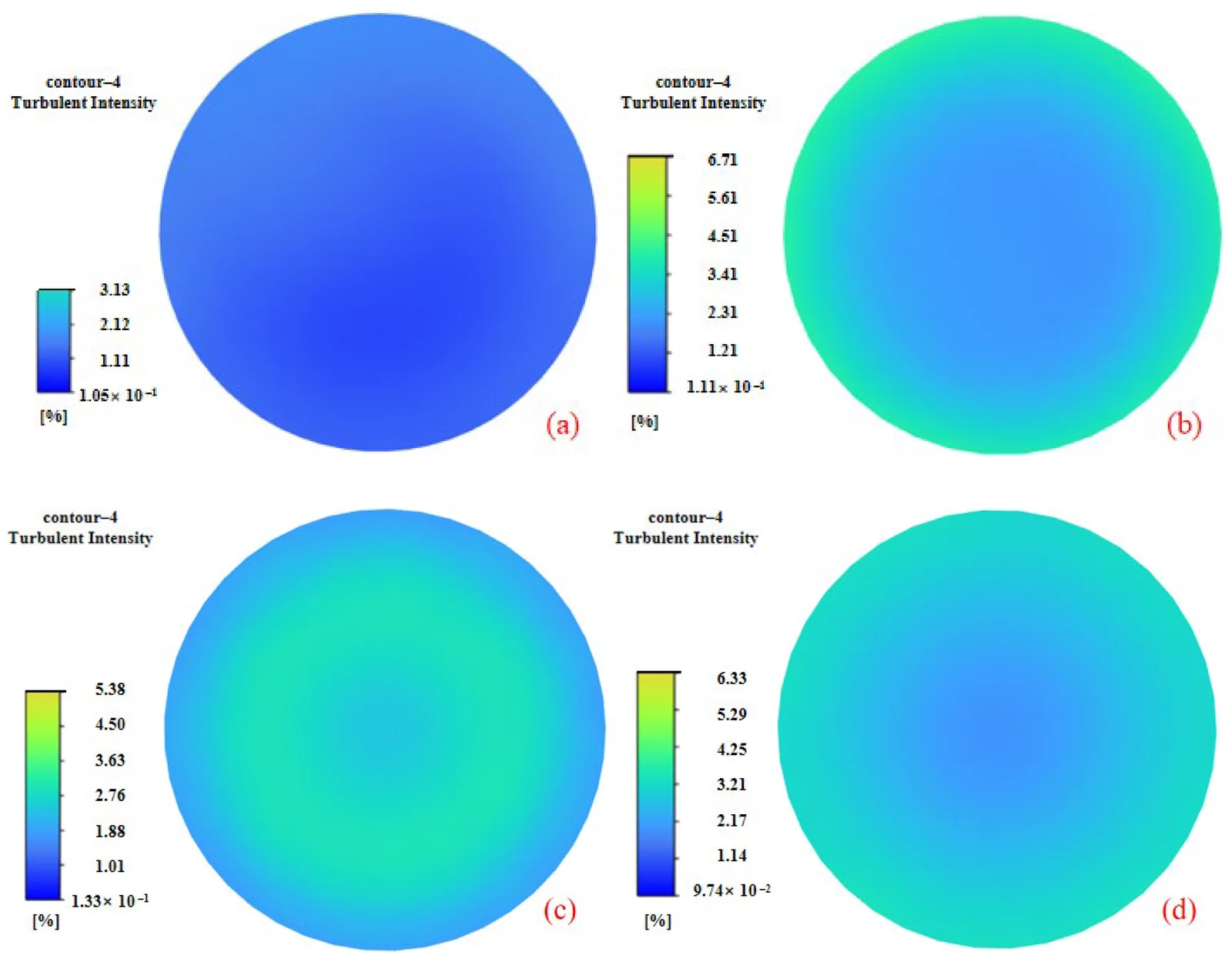
Cloud maps of the turbulence intensity distribution at the outlets of the four types of nozzles. (**a**) Cylindrical type (**b**) Tapered type (**c**) Tapered-diffuser type (**d**) Tapered-cylindrical type.

**Figure 8:**
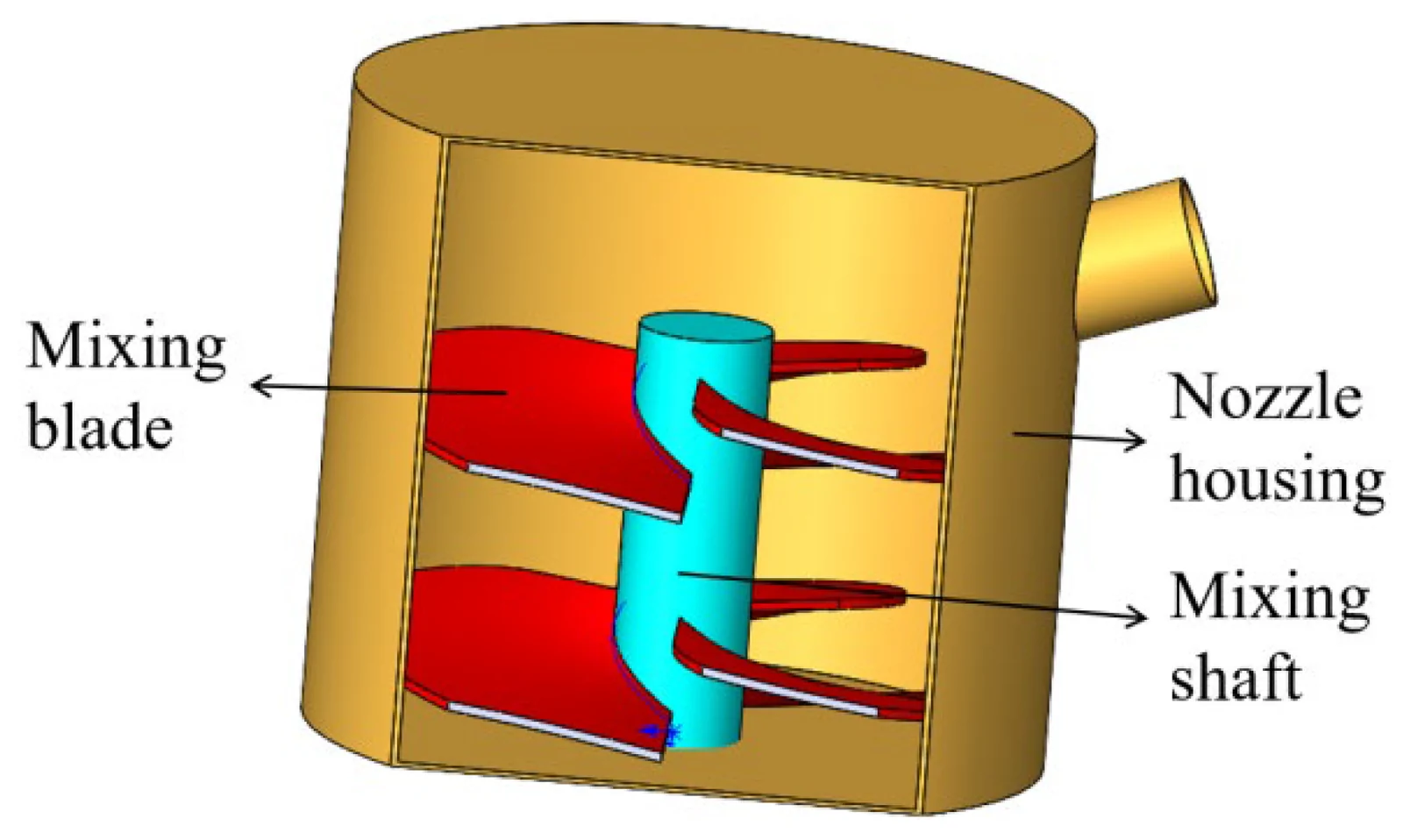
Simplified 3D model of the nozzle mixing section (mixing zone).

**Figure 9:**
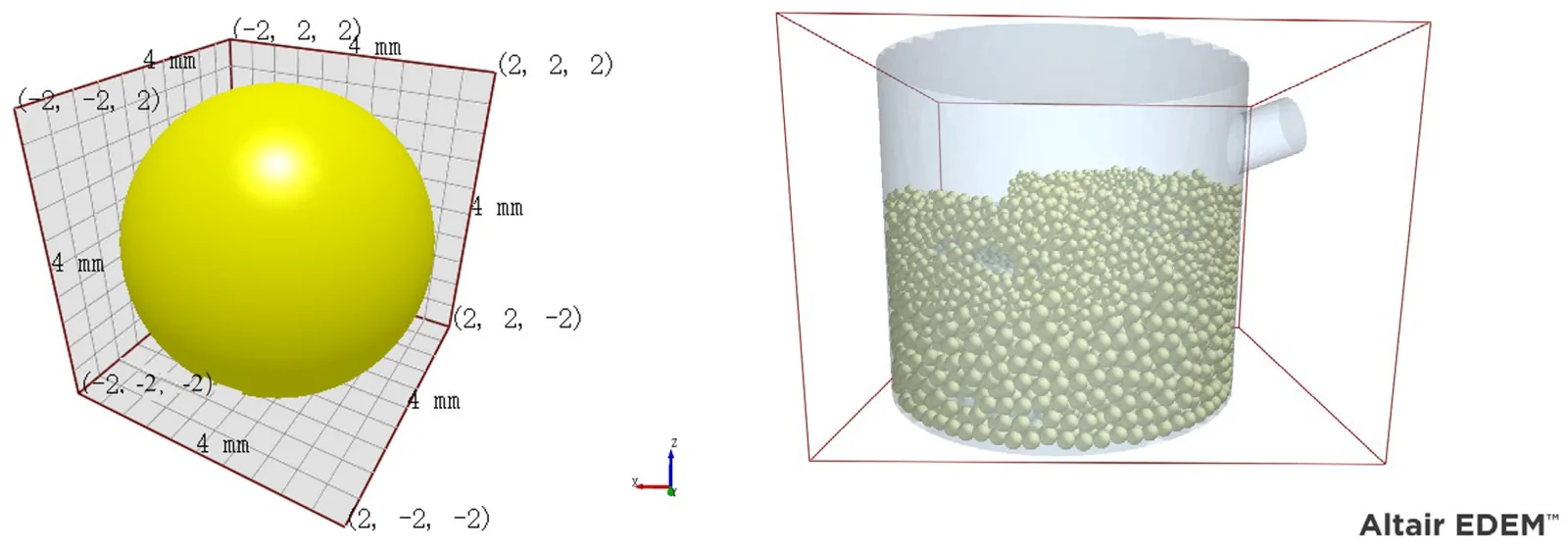
Generation of stirring particles.

**Figure 10:**
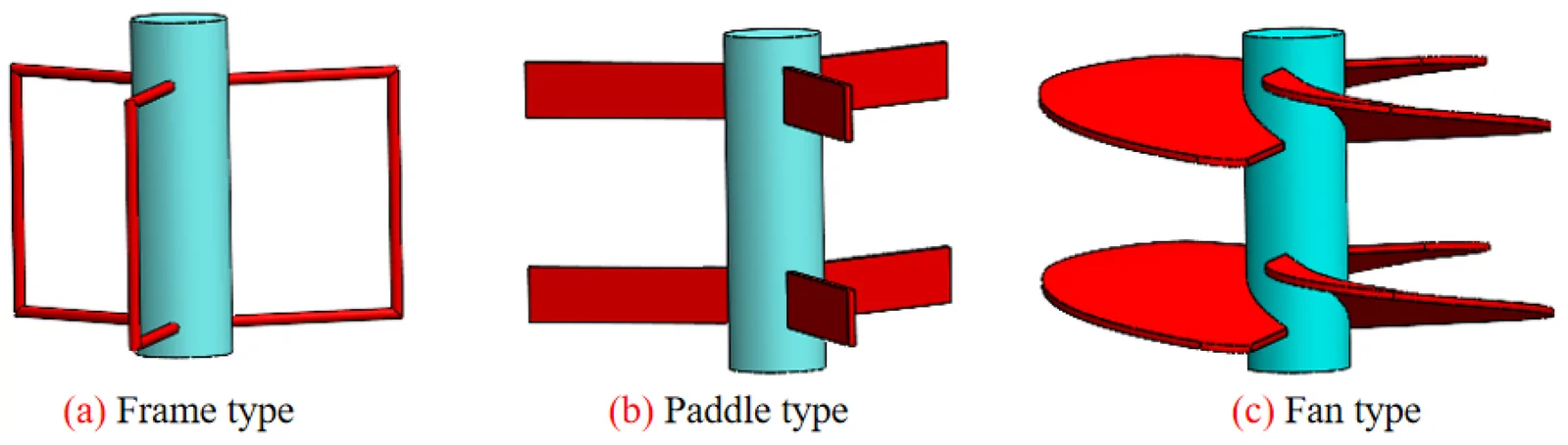
Common mixer types.

**Figure 11:**
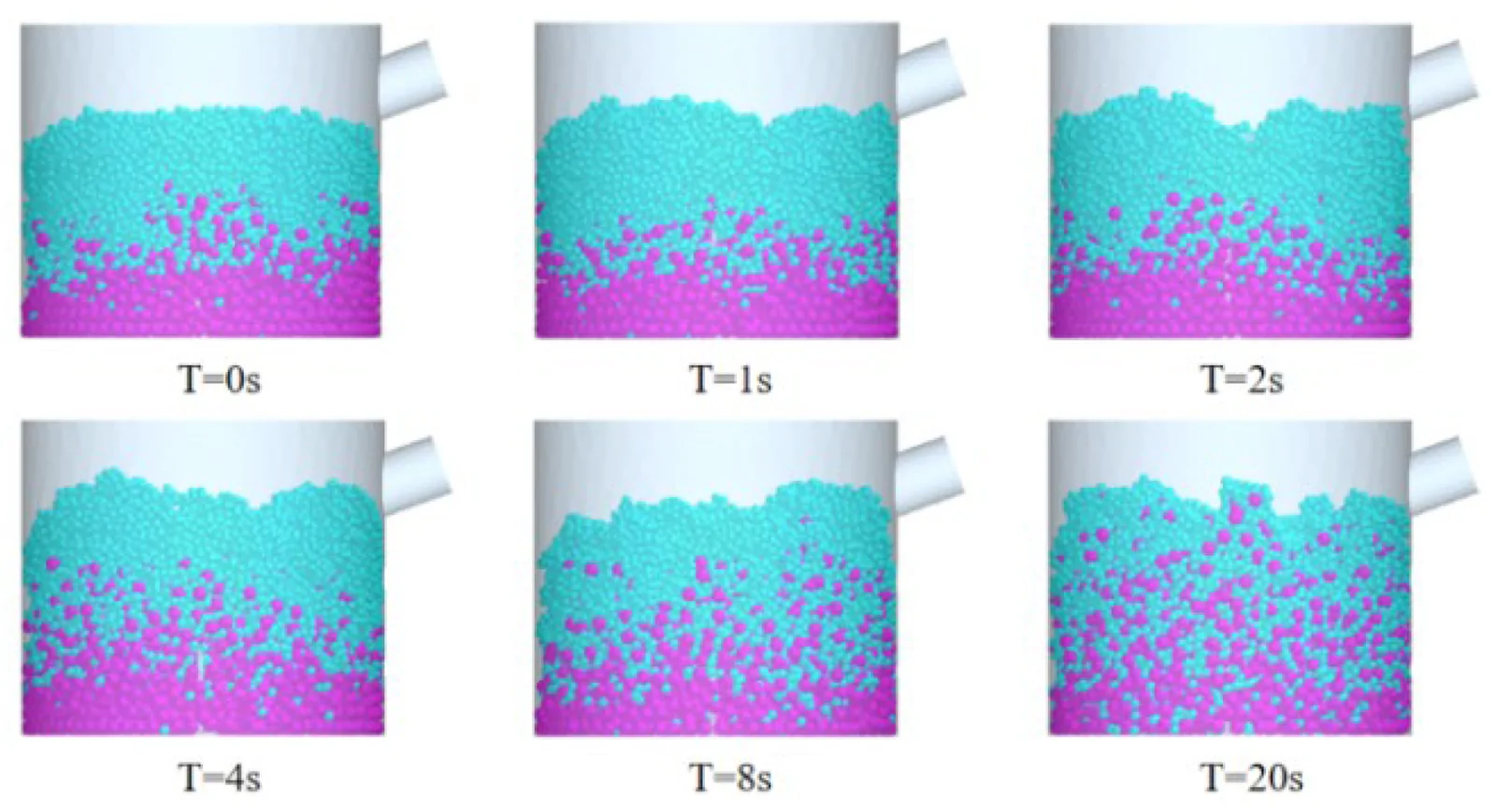
Simulation process for box particle stirring, T = 0–20 s.

**Figure 12:**
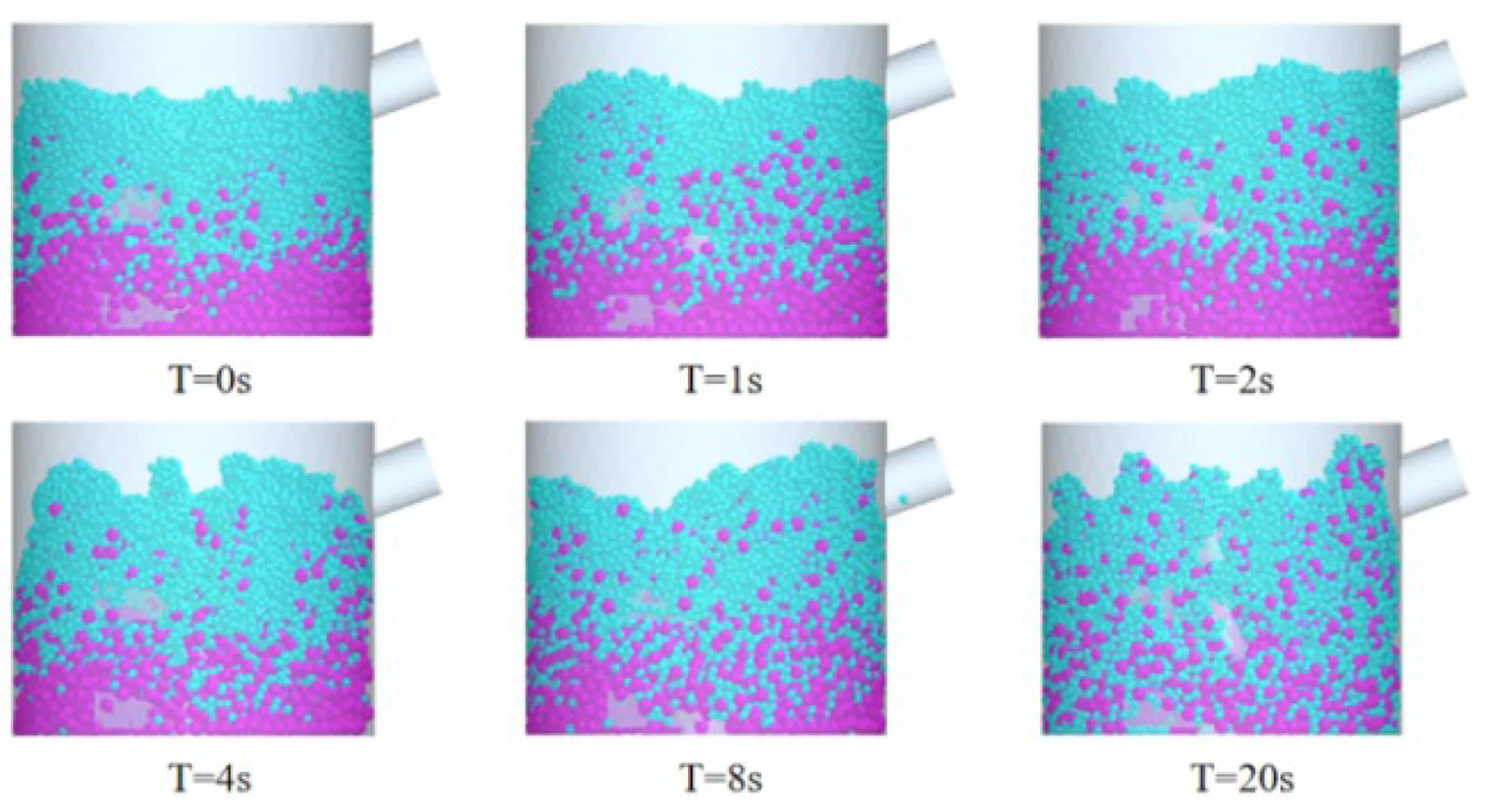
Simulation process for paddle particle stirring, T = 0–20 s.

**Figure 13:**
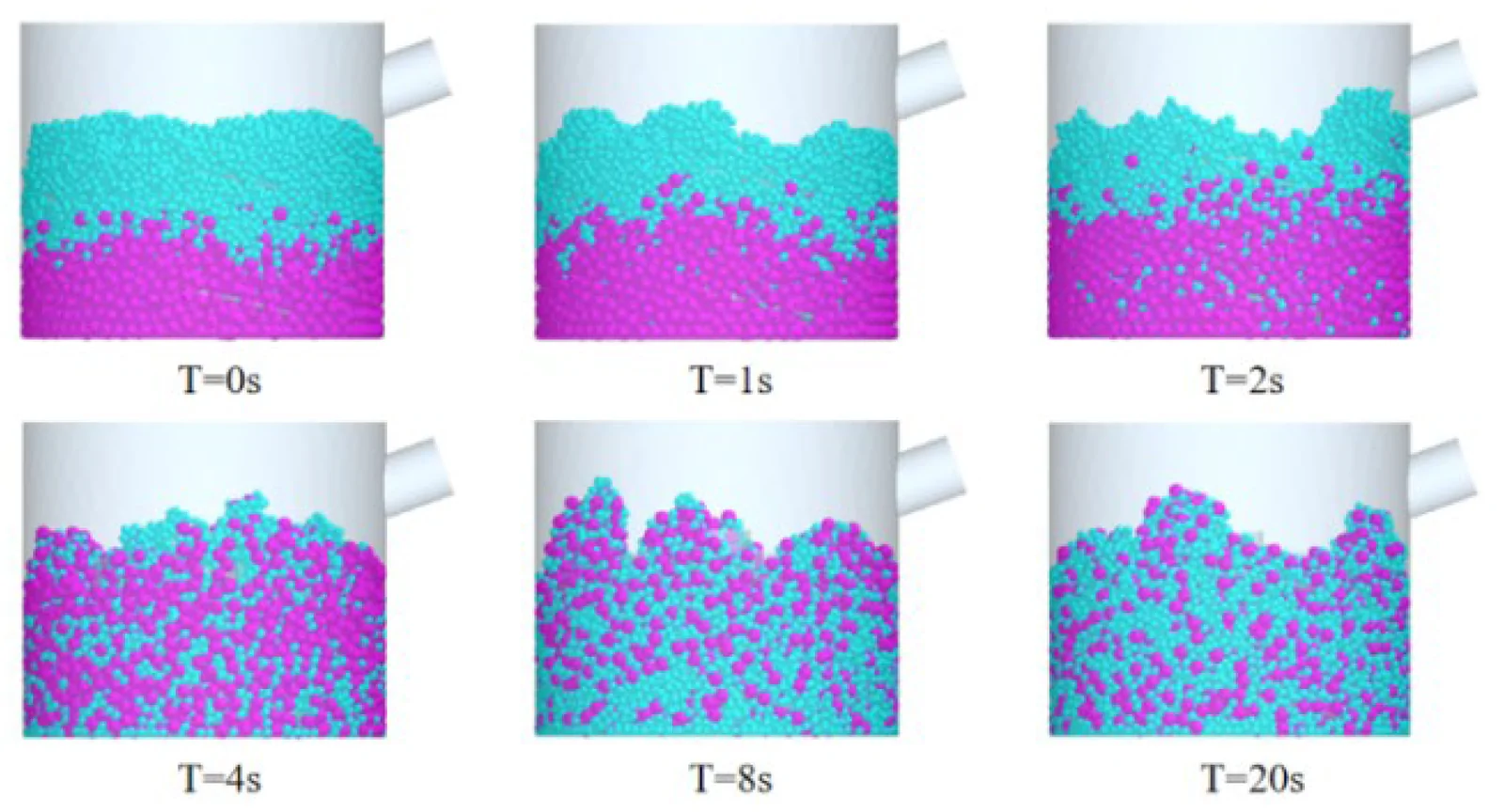
Simulation process for fan-type particle stirring, T = 0–20 s.

**Figure 14:**
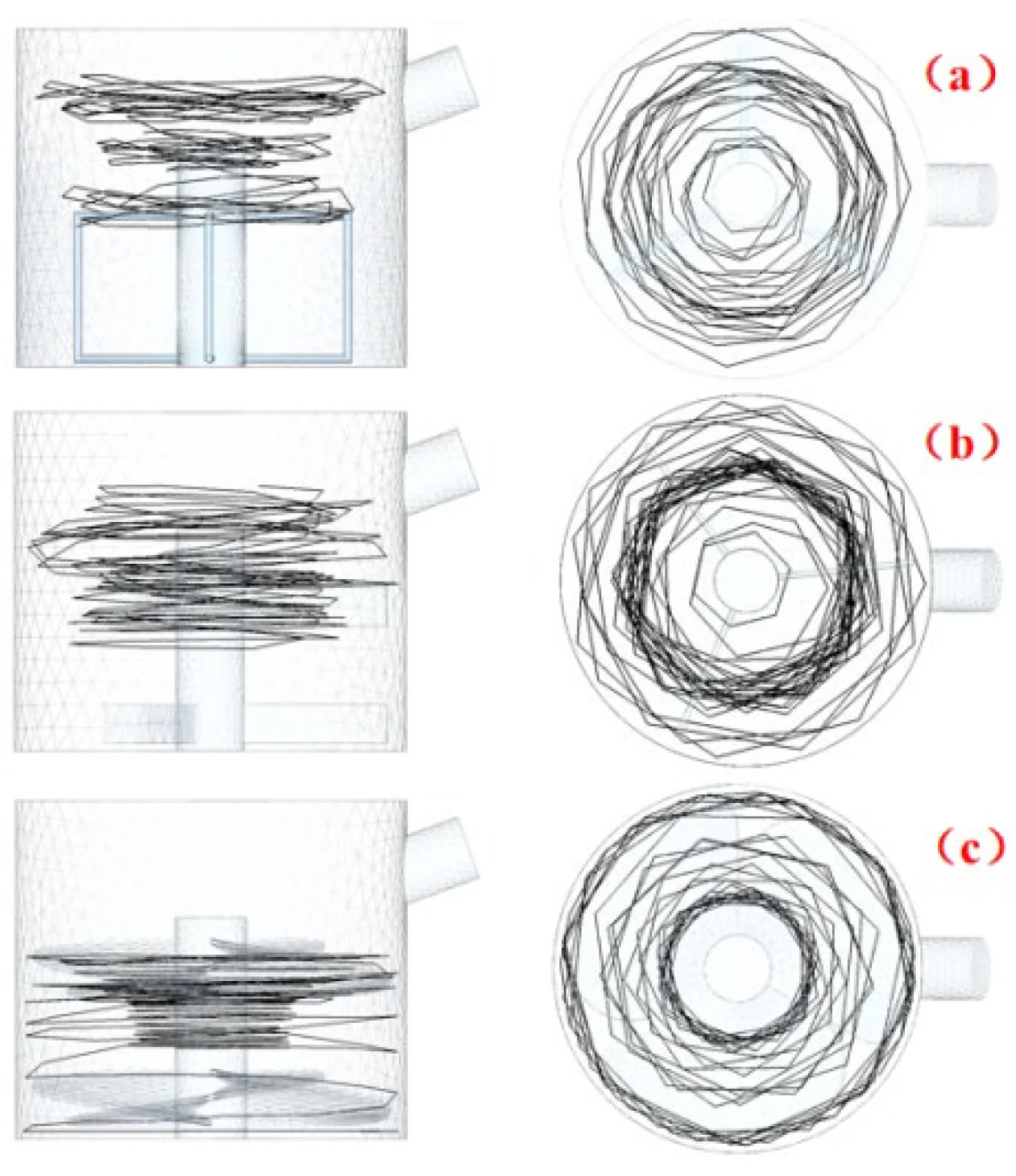
Front and top views of single particle trajectory at T = 20 s. (**a**) frame mixing; (**b**) paddle mixing; (**c**) fan mixing.

**Figure 15:**
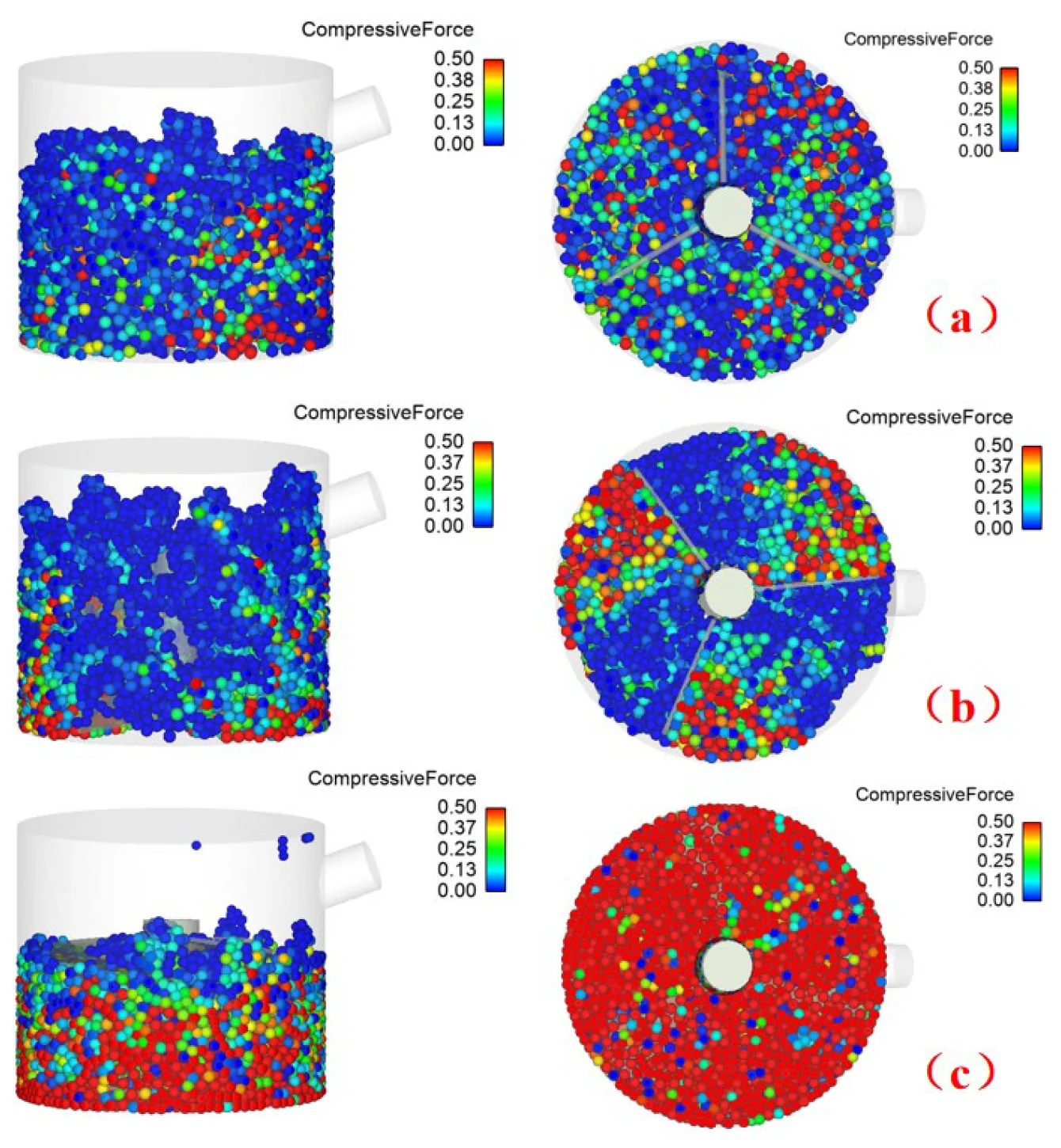
Particle pressure at T = 20 s: (**a**) frame mixing; (**b**) paddle mixing; (**c**) fan-type mixing.

**Figure 16:**
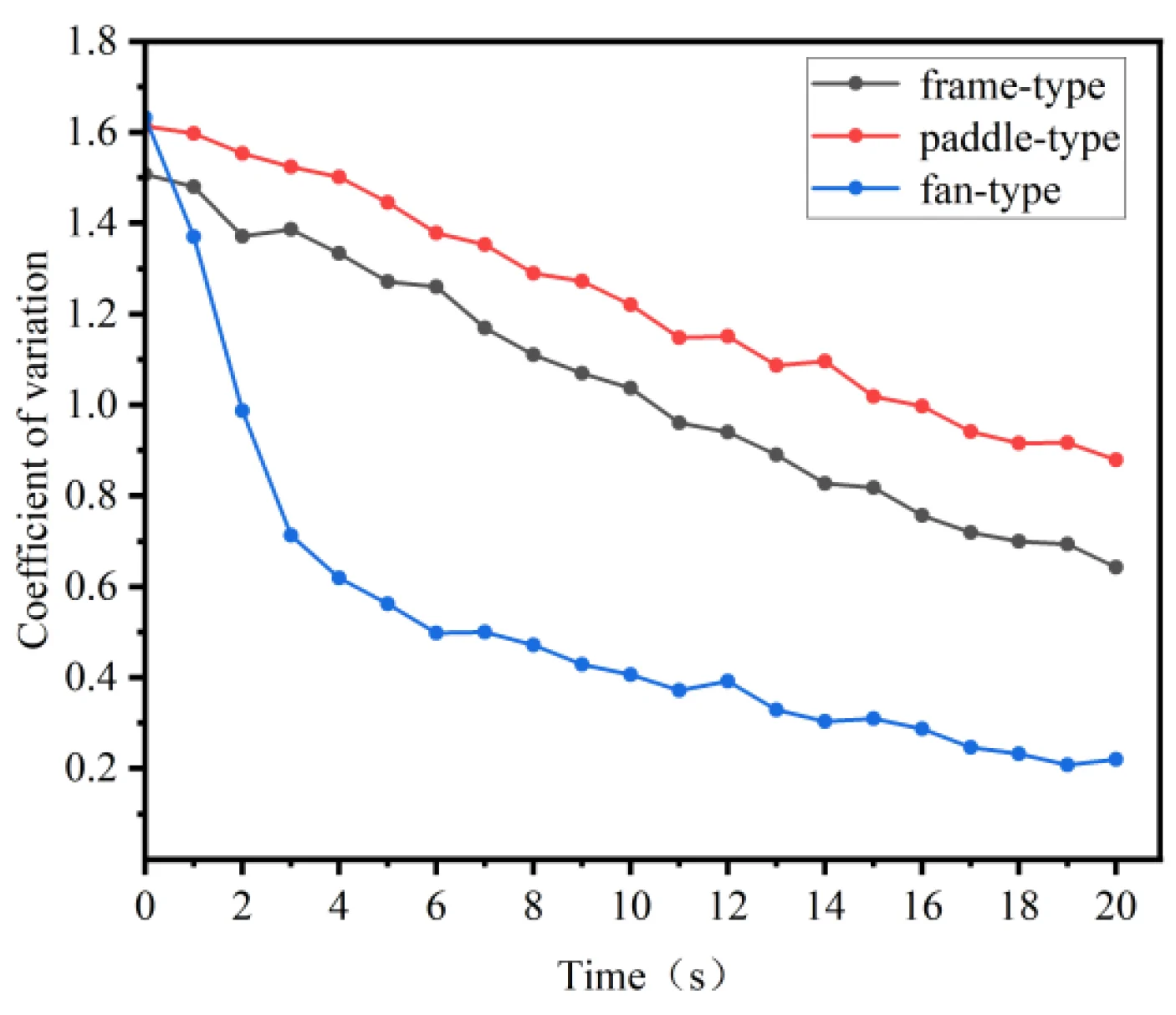
Discrete coefficient diagram of box, paddle, and fan mixing, T = 0–20 s.

**Figure 17:**
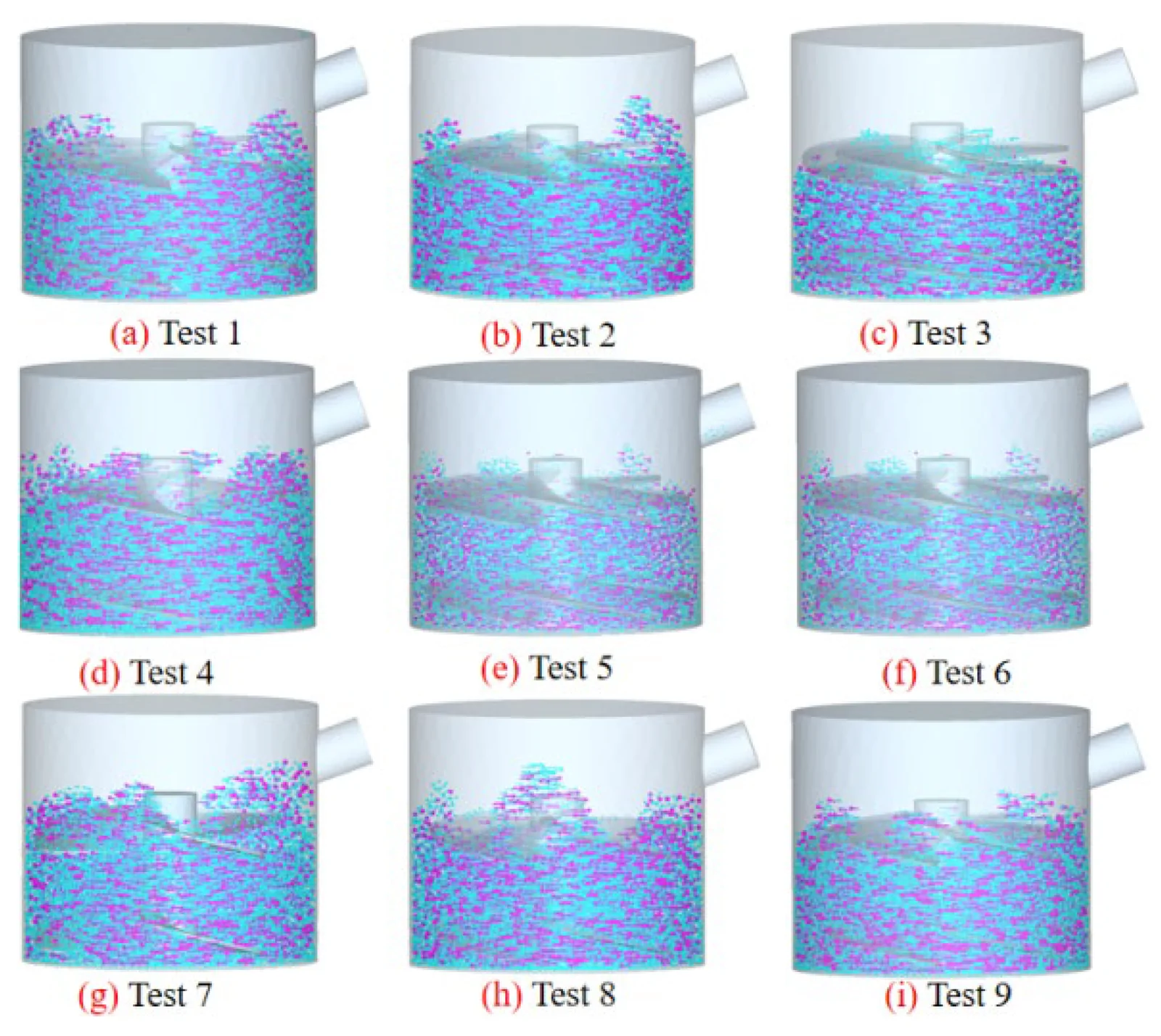
Vector diagram of the concrete mixing particles at T = 10 s.

**Figure 18:**
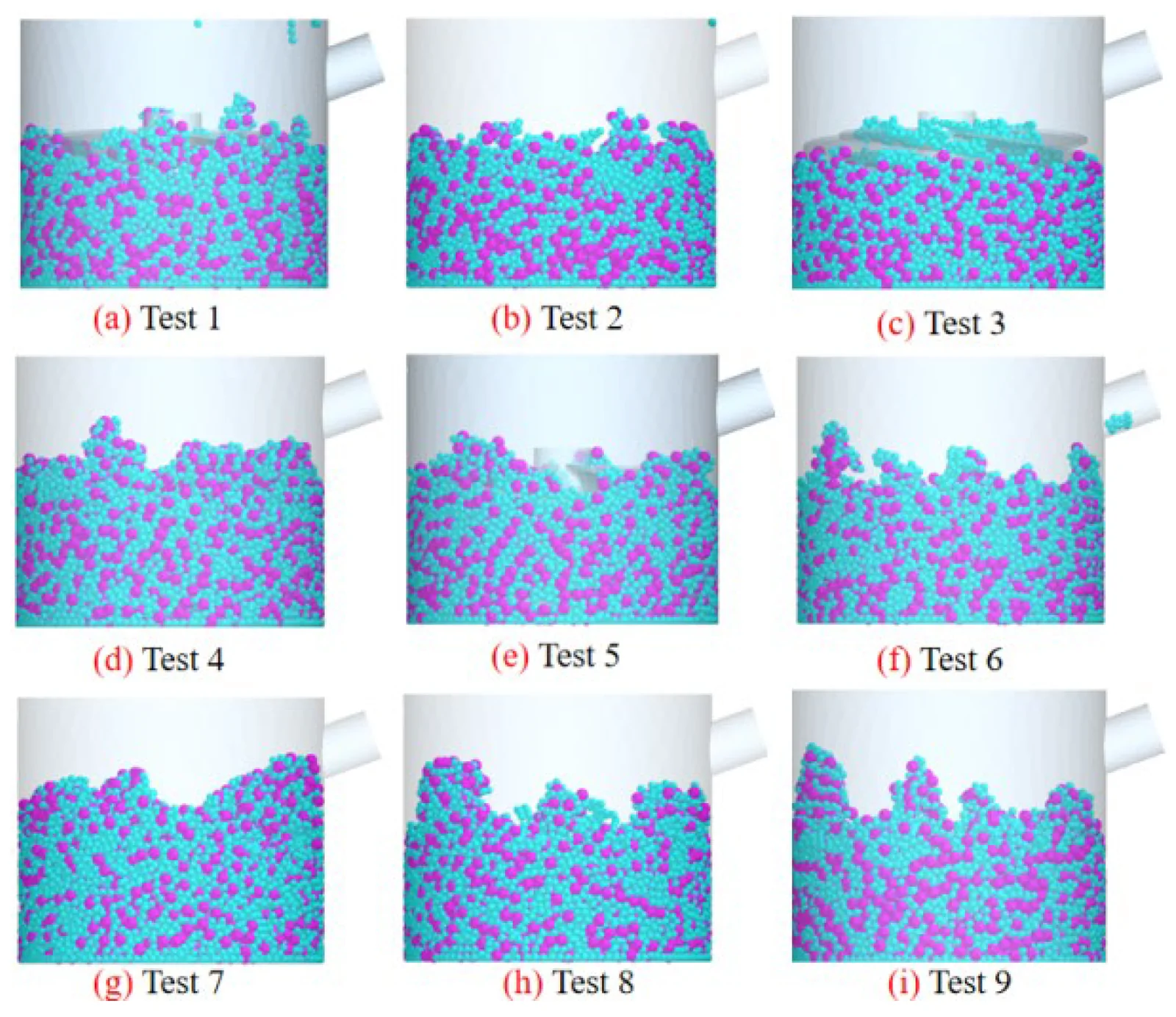
Distribution of the concrete mixing particles at t = 20 s.

**Figure 19:**
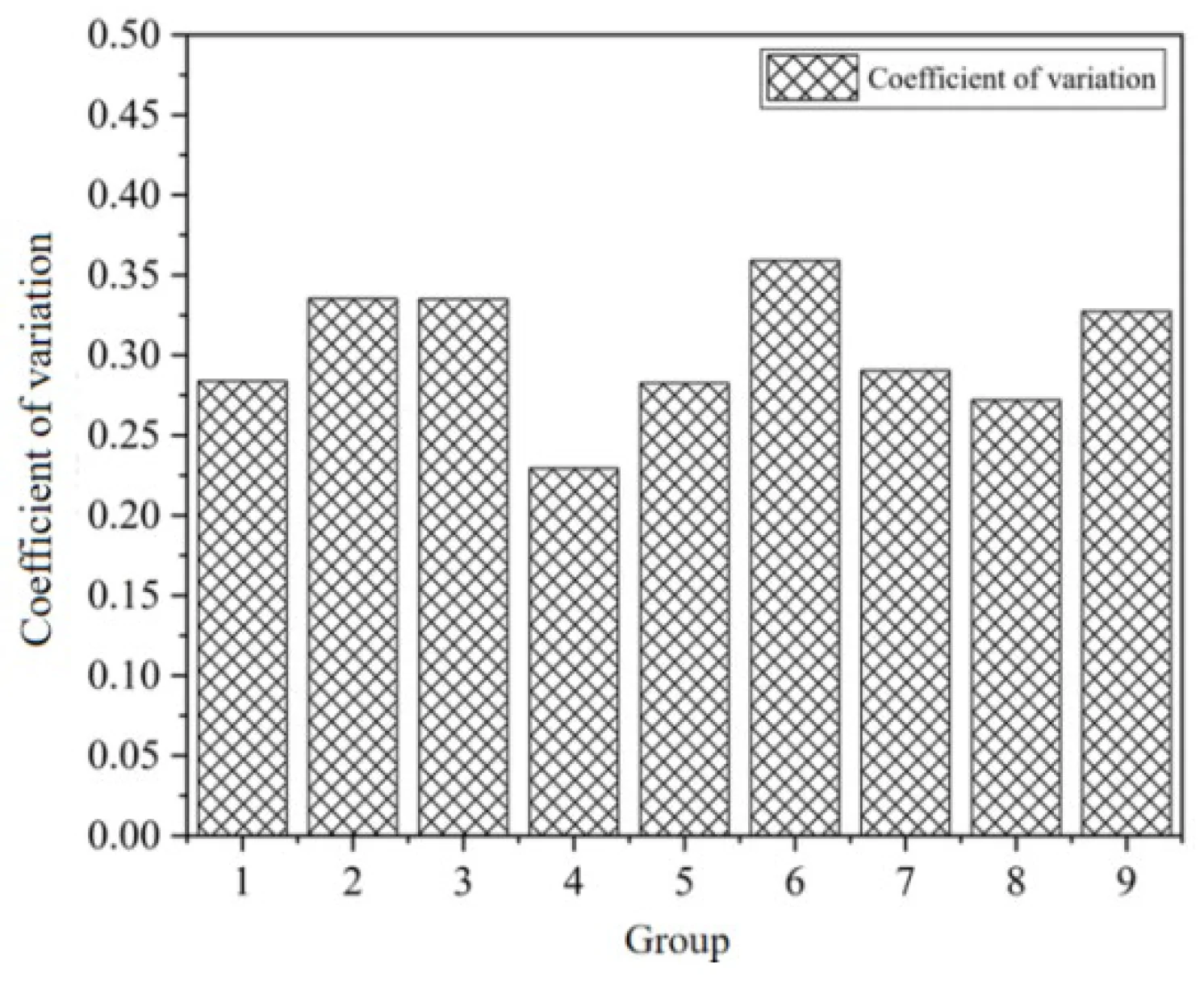
Discrete coefficients for each experimental group.

**Figure 20:**
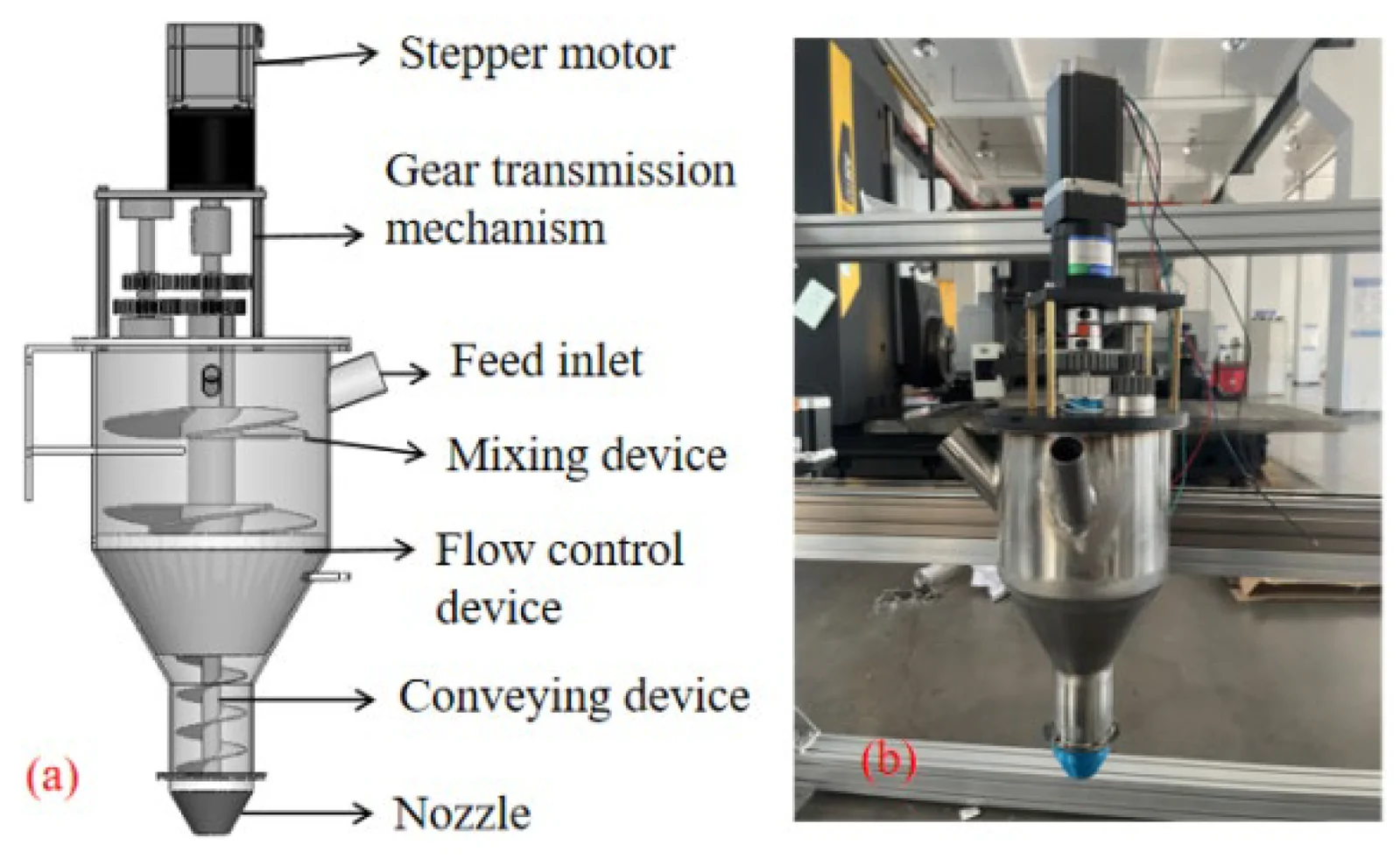
Principle prototype: (**a**) 3D structural diagram; (**b**) actual photograph.

**Figure 21:**
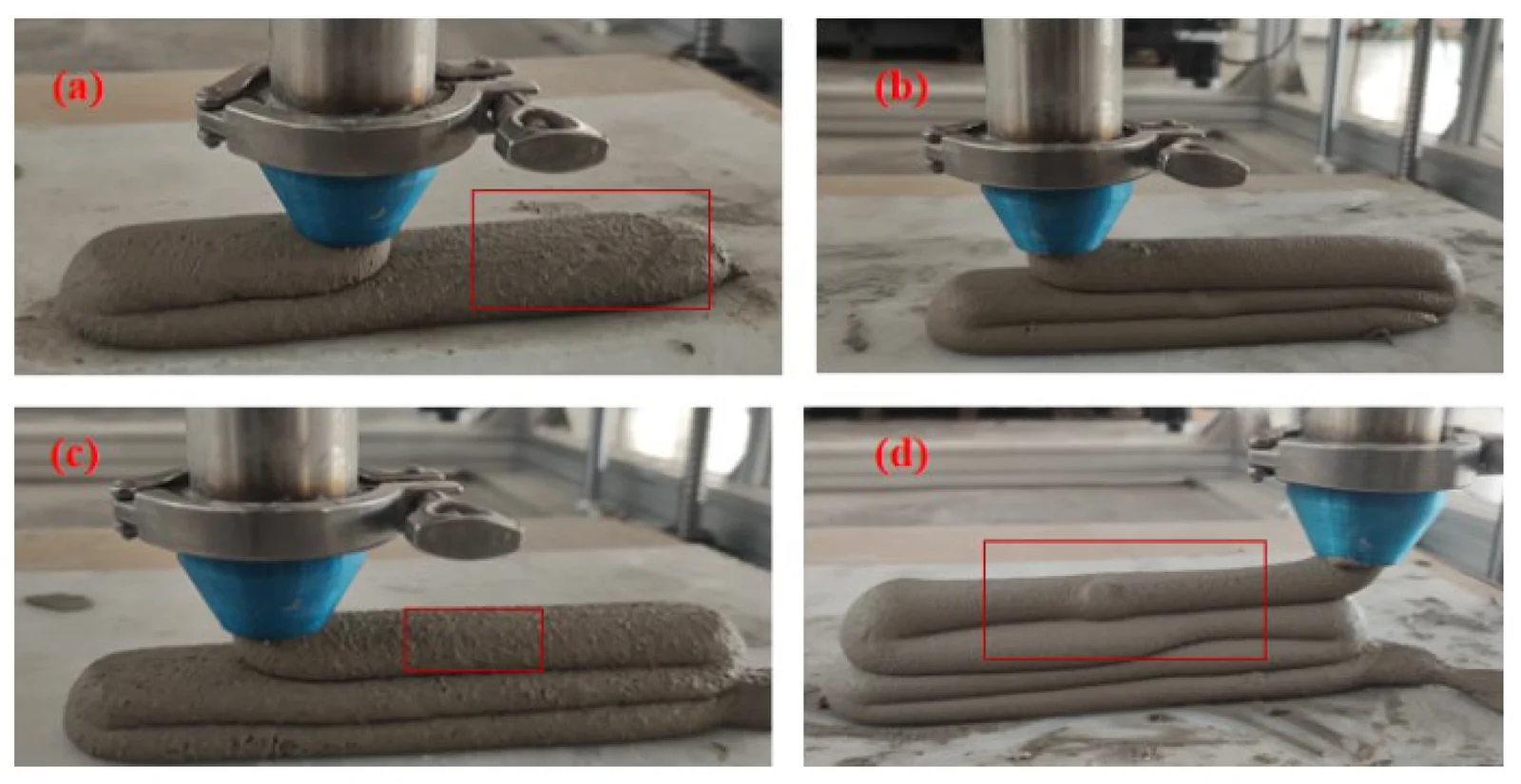
Comparison of the surface forming quality of specimens with four different printing speeds: (**a**) 5 mm/s; (**b**) 10 mm/s; (**c**) 15 mm/s; (**d**) 20 mm/s. The red frames are included in the text to differentiate the forming quality caused by varying printing speeds.

**Figure 22:**
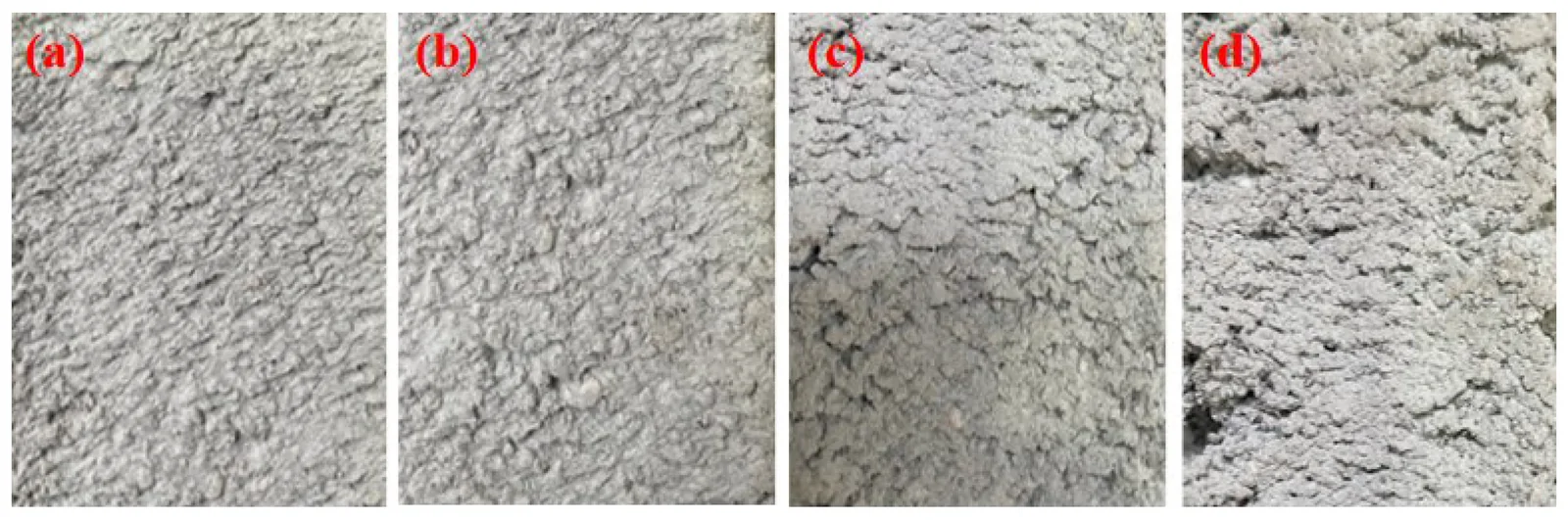
Surface roughness of specimens cured for seven days at four different printing speeds. (**a**) 5 mm/s; (**b**) 10 mm/s; (**c**) 15 mm/s; (**d**) 20 mm/s.

**Figure 23:**
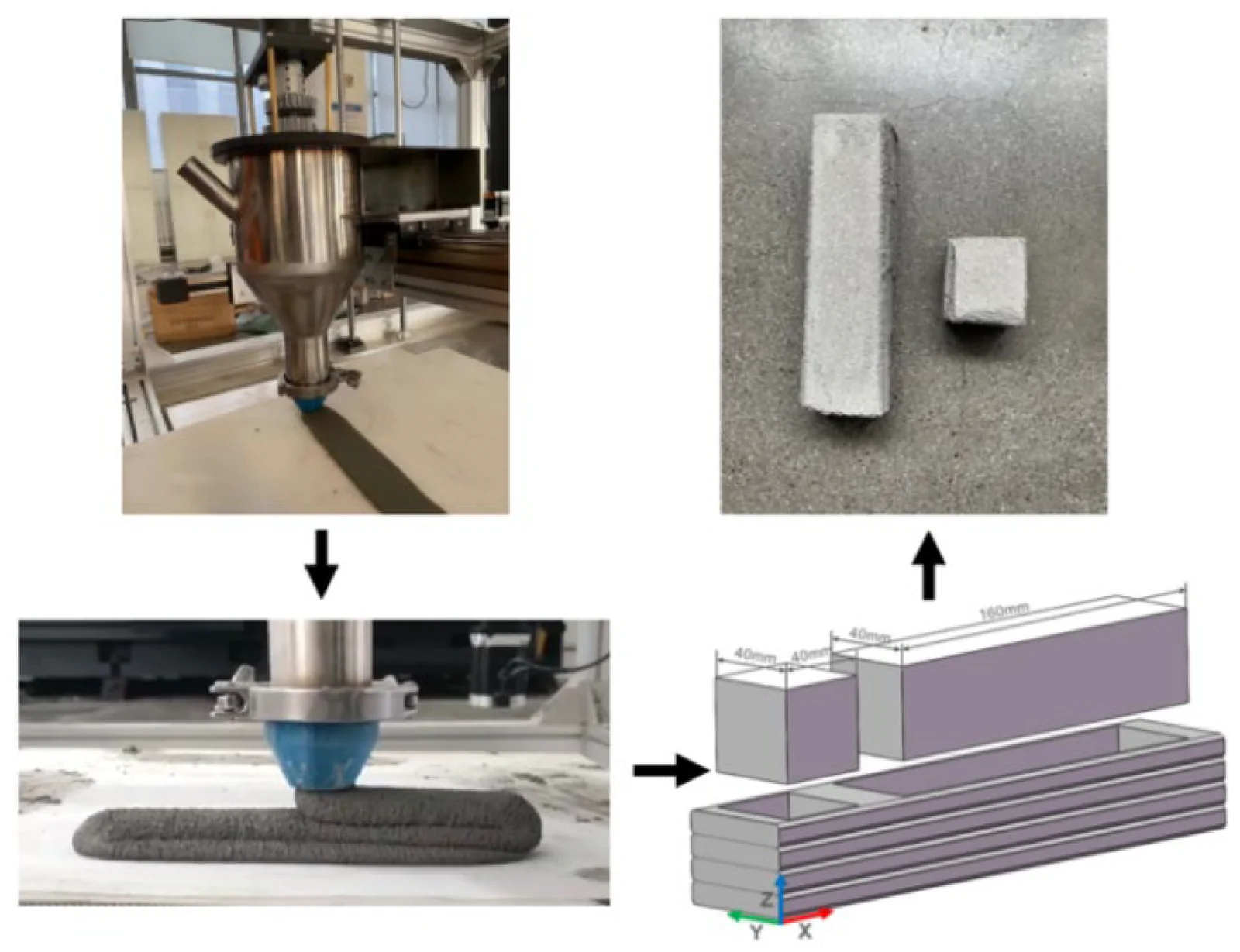
Preparation process for the concrete 3d printing test blocks.

**Figure 24:**
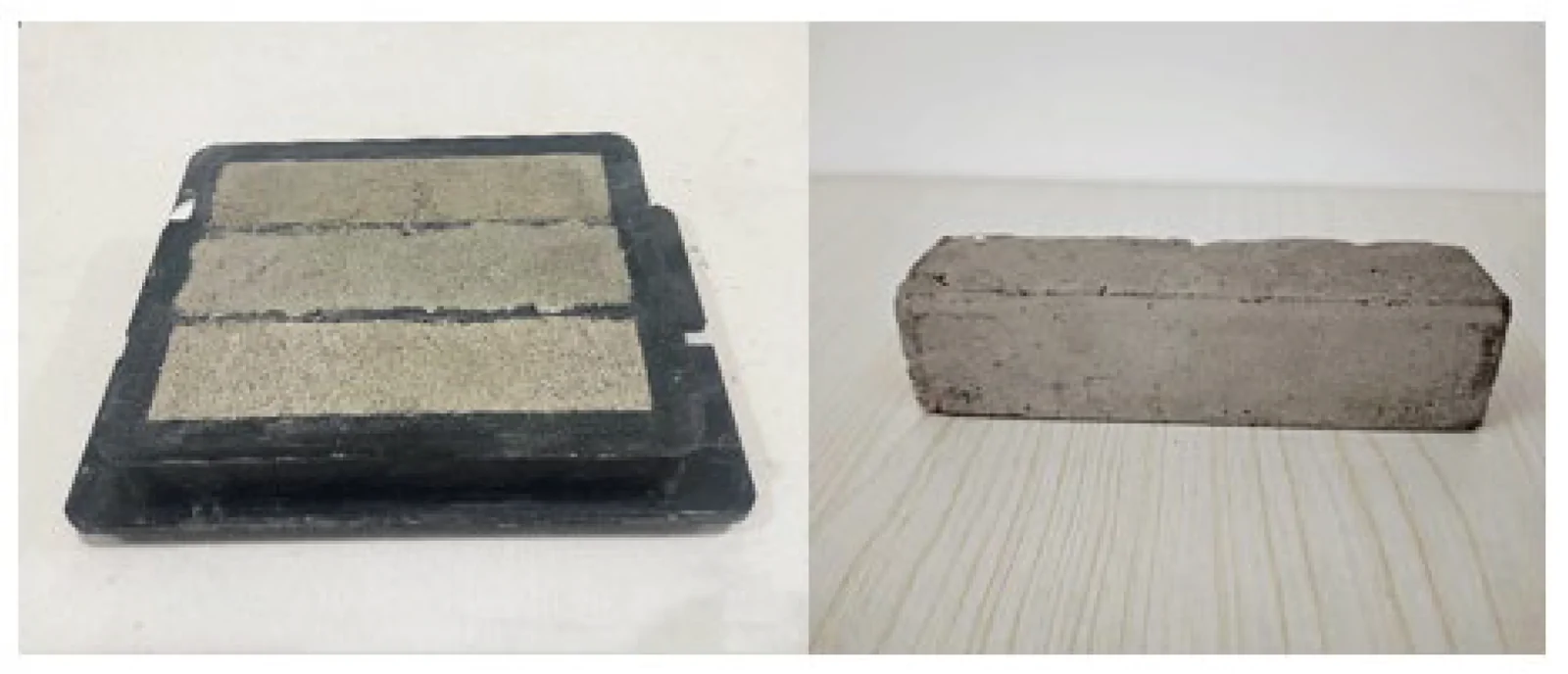
Pouring test mold and test block.

**Figure 25:**
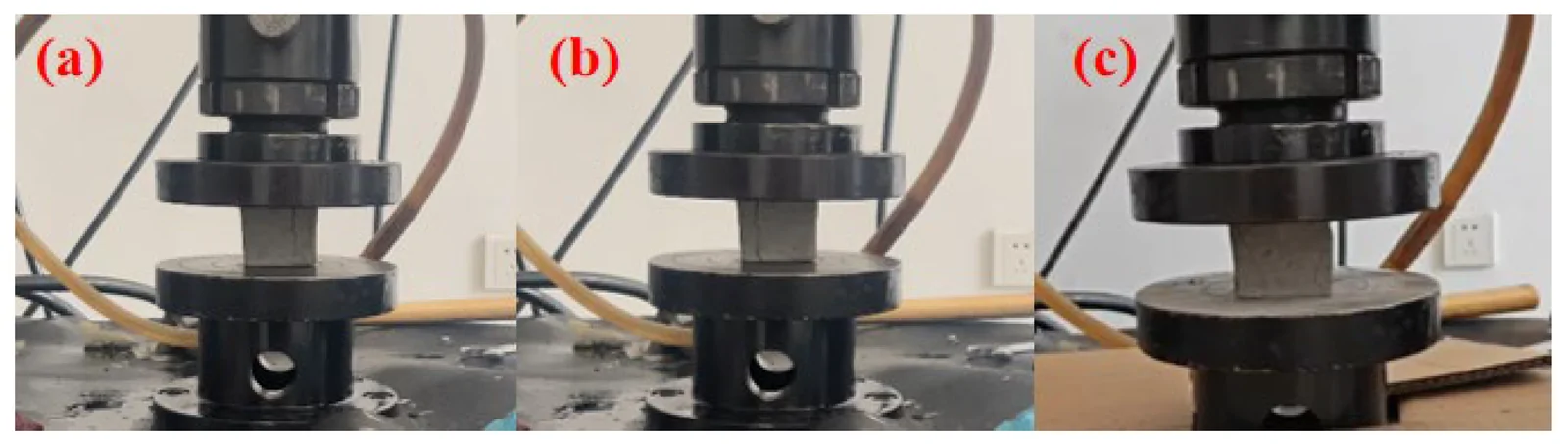
Three sets of compressive tests on concrete specimens of the same age group: (**a**) A1 ordinary printing; (**b**) A2 fast-hardening printing; (**c**) A2-1 pouring.

**Figure 26:**
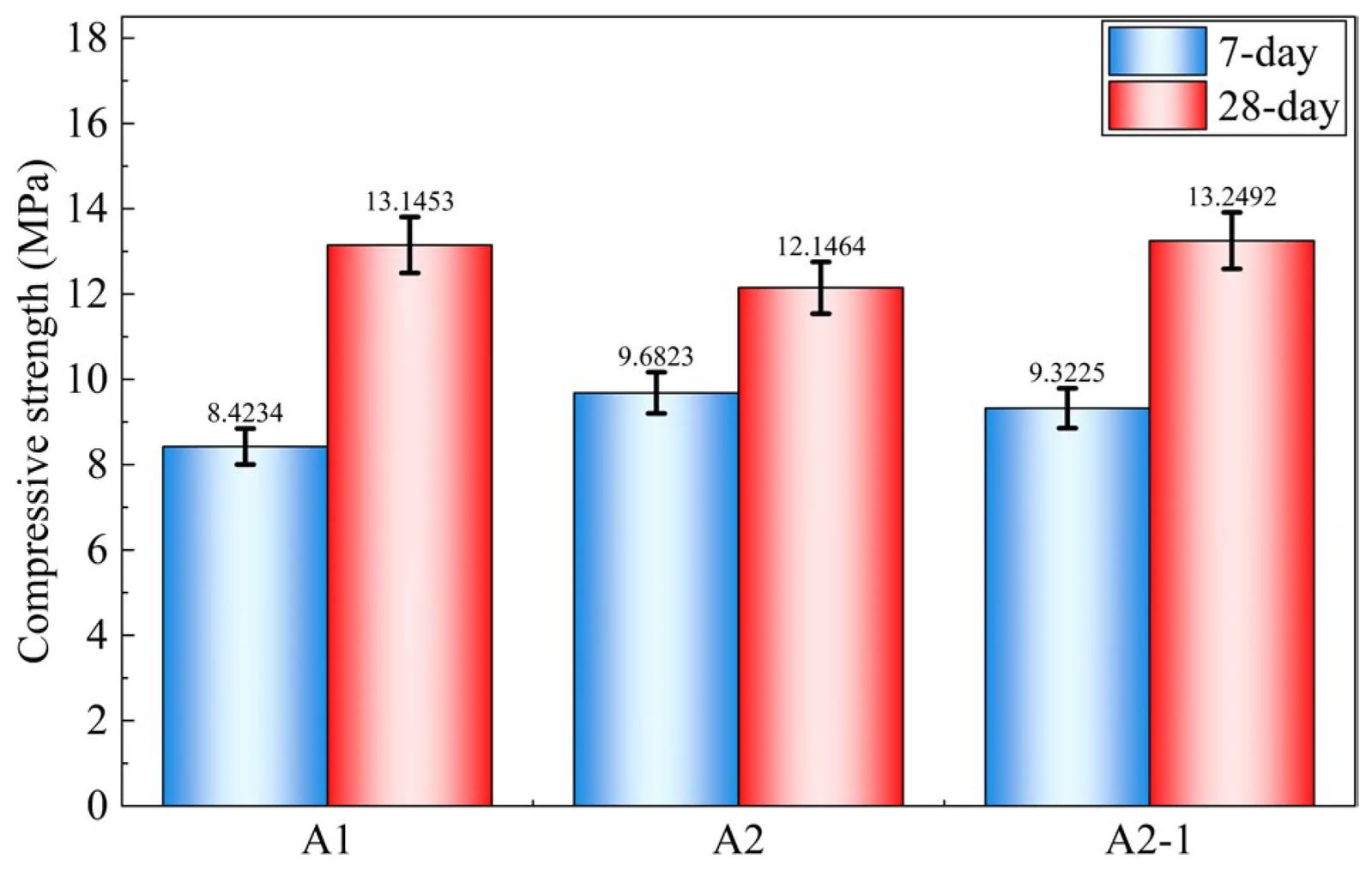
Comparison of the compressive strength performances of three groups of concrete test blocks of the same age group.

**Figure 27:**
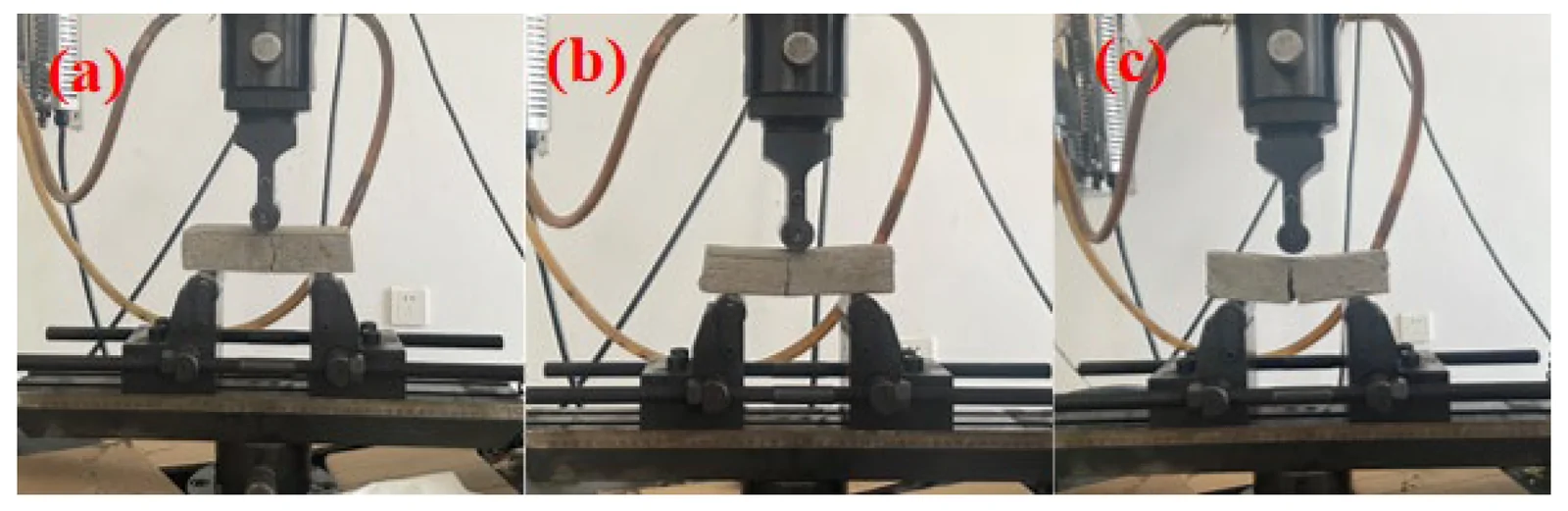
Three sets of concrete test blocks of the same age for flexural testing: (**a**) A2 regular printing; (**b**) A2 fast-hardening printing; (**c**) A2-1 pouring.

**Figure 28:**
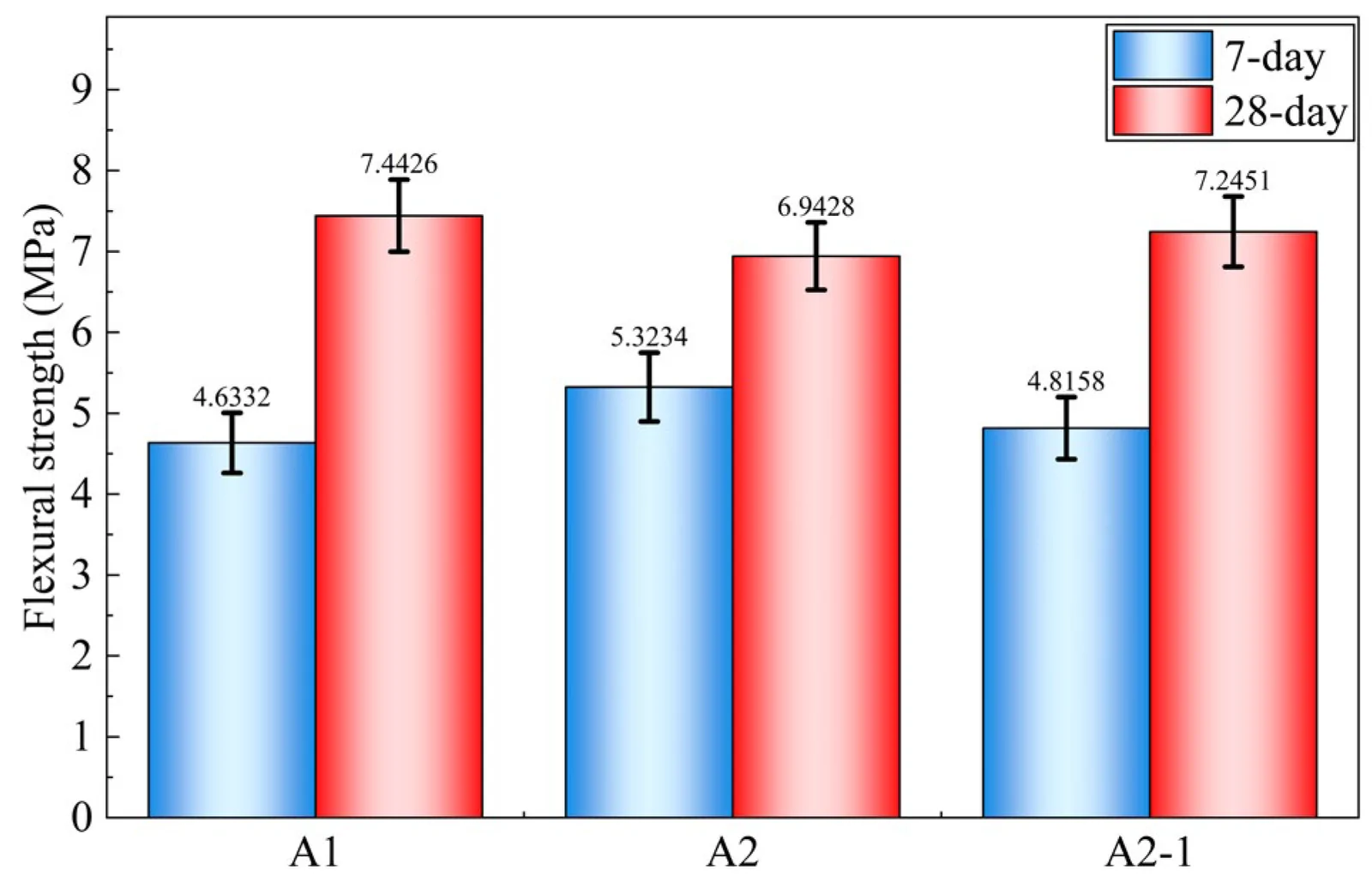
Comparison of the flexural strength performances of three groups of concrete test blocks of the same age group.

**Table 1:** Screw structure parameters.

Parameter	Value	Unit
Outer diameter of mixing barrel	150	
Height, H	280	
Diameter of powder inlet tube	25	
Diameter of liquid inlet tube	15	
Extrusion tube diameter	47	
Screw outer diameter, *D*	45	mm
Aspect ratio	3.5	-
Channel depth, *H*_3_	16	mm
Screw pitch, *S*	45	mm
Flight width, *e*	3	mm
Helix angle, *φ*	17.7°	degrees

**Table 2:** Parameters of the flow properties fitted by the four models.

Rheological Model	Rheological Equation	Coefficient of Determination (R^2^)	Number of Data Points
Bingham model	$\tau =\tau_{0}+\mu \gamma$	0.828	15
Power-law model	$\tau =Ky^{n}$	0.951	
Modified Bingham model	$\tau =\tau_{0}+\mu \dot{\gamma }+c\gamma^{2}$	0.996	
Herschel–Bulkley	$\tau =\tau_{0}+K\cdot y^{n}$	0.978	

**Table 3:** Material properties.

Material	Poisson’s Ratio	Shear Modulus (Pa)	Density (kg·m^−3^)
Coarse aggregate	0.3	2 × 109	2600
Fine aggregate	0.25	1 × 108	2100
Steel	0.3	1 × 1010	7850

**Table 4:** Contact parameters and JKR surface energy of concrete component materials.

	Aggregate–Aggregate	Cement–Cement	Aggregate–Cement	Aggregate–Steel	Cement–Steel
Coefficient of Restitution	0.15	0.02	0.05	0.2	0.05
Coefficient of Static Friction	0.32	0.13	0.06	0.3	0.09
Coefficient of Rolling Friction	0.12	0.16	0.08	0.5	0.12
JKR/(J·m^−2^)	0.9	6.4	10.3	0.8	2.3

**Table 5:** Level of mixing test factors.

Level	Leaf Angle A/°	Number of Blades B/Each	Stirring Speed C/rpm
1	35	3	100
2	40	4	120
3	45	5	140

**Table 6:** Orthogonal experimental scheme table.

Test Group Number	A	B	C
1	1	1	1
2	1	2	2
3	1	3	3
4	2	1	2
5	2	2	3
6	2	3	1
7	3	1	3
8	3	2	1
9	3	3	2

**Table 7:** Results from the orthogonal experiment.

Test Group Number	A	B	C	Coefficient of Variation
1	1	1	1	0.2837
2	1	2	2	0.3353
3	1	3	3	0.3350
4	2	1	2	0.2294
5	2	2	3	0.2825
6	2	3	1	0.3590
7	3	1	3	0.2902
8	3	2	1	0.2718
9	3	3	2	0.3273

**Table 8:** Range of discrete coefficients.

	A			B			C		
Index	kA1	kA2	kA3	kB1	kB2	kB3	kC1	kC2	kC3
CV	0.318	0.290	0.296	0.268	0.297	0.340	0.305	0.297	0.303
Range	0.028			0.072			0.008		

**Table 9:** Physical and mechanical performance indicators of SAC cement materials.

Type	Specific Surface Area (m^2^·kg^−1^)	Setting Time (Min)		1-Day Intensity (MPa)		3-Day Intensity (MPa)	
		Initial Setting	Final Set	Flexural	Compression Resistance	Flexural	Compression Resistance
R.SAC42.5	497	3	5	6.1	37.2	6.5	45.1

**Table 10:** Basic mix proportions of the cementitious materials for each group (unit: kg/m^3^).

Number	SAC	SAND	MP	FA	HPMC	WR	Li_2_CO_3_	Water
A1	550	440	225	225	1.25	37.5	0	165
A2	550	440	225	225	1.25	37.5	2.25	165
A2-1	550	440	225	225	1.25	37.5	2.25	165

**Table 11:** Monitoring data from the 1 h continuous printing test.

Time (Min)	Extrusion Pressure (MPa)	Screw Torque (N·m)	Extrusion Mass Flow Rate (g/s)	Extruded Filament Width (mm)	Status Description
0	0.52	2.4	28.6	25.1	Start (t = 0)
10	0.53	2.4	28.4	25.2	Normal operation
20	0.54	2.5	28.5	25.0	Normal operation
30	0.55	2.6	28.2	25.1	Midway check, no abnormality
40	0.56	2.7	28.0	25.2	Normal operation
50	0.57	2.8	27.8	25.3	Normal operation
60	0.61	3.0	27.0	25.6	End, no clogging

## Data Availability

The original contributions presented in this study are included in the article. Further inquiries can be directed to the corresponding author.

## Nomenclature

*E*	Equivalent Young’s modulus
*R*	Equivalent radius
*γ*	Surface energy

## References

[1] Paul S.C., Van Zijl G.P., Tan M.J., Gibson I. (2018). A review of 3D concrete printing systems and materials properties: Current status and future research prospects. Rapid Prototyp. J..

[2] Choong Y.Y.C., Tan H.W., Patel D.C., Choong W.T.N., Chen C.H., Low H.Y., Tan M.J., Patel C.D., Chua C.K. (2020). The global rise of 3D printing during the COVID-19 pandemic. Nat. Rev. Mater..

[3] Zhang B., Kang J., Li J., Liang J., Wang J. (2022). Evaluation of interface rapid bond strength between normal concrete and ternary system fast setting and rapid hardening self-compacting concrete. Constr. Build. Mater..

[4] Babich Y., Filipchuk S., Karavan V., Sobczak-Piąstka J. (2019). Research of basic mechanical and deformative properties of high-strength fast-hardening concretes. Scientific Session on Applied Mechanics.

[5] Bolina F.L., Henn A.S., Silva D.B., Pachla E.C. (2025). Numerical Evaluation of Modified Mortar Coatings for Thermal Protection of Reinforced Concrete and Steel Structures Under Standardized Fire Exposure. Coatings.

[6] Han J., Yan P. (2011). Effect of lithium carbonate on hydration characteristics and strength development of sulfoaluminate cement. J. Build. Mater..

[7] Cheikh E.K., Rémond S., Khalil N., Aouad G. (2017). Numerical and experimental studies of aggregate blocking in mortar extrusion. Constr. Build. Mater..

[8] Cao G., Zhang H., Tan Y., Wang J., Deng R., Xiao X., Wu B. (2015). Study on the effect of coarse aggregate volume fraction on the flow behavior of fresh concrete via DEM. Procedia Eng..

[9] Pantaleev S., Yordanova S., Janda A., Marigo M., Ooi J.Y. (2017). An experimentally validated DEM study of powder mixing in a paddle blade mixer. Powder Technol..

[10] Yang L., Sepasgozar S.M.E., Shirowzhan S., Kashani A., Edwards D. (2023). Nozzle criteria for enhancing extrudability, buildability and interlayer bonding in 3D printing concrete. Autom. Constr..

[11] Niu H., Yu B., Hao J. (2025). CFD-based flow field analysis of spiral nozzles in 3D concrete printing. Discov. Appl. Sci..

[12] Pu X., Zhang R., Du J., Zhang H. (2025). Design and simulation of spiral blade in the nozzle for the extrusion-based 3D printing concrete. Mech. Based Des. Struct. Mach..

[13] Liu J., Ou K. (2020). Simulation analysis of planetary concrete mixing equipment based on EDEM. Constr. Mach..

[14] Wang D., Yu P., Ding Y., Yang W. (2023). Numerical simulation of extrusion flow process of natural polymer in laminated channel. China Plast..

[15] Wang Y., Ren C., Yan M., Ao C. (2024). Blockage mechanism analysis and optimization design of 3D concrete printhead. J. Sustain. Cem.-Based Mater..

[16] Zhang X., Chang S., Zhang Y., Zhou Y. (2012). Influence of particle group characteristics of lightweight aggregate on interfacial properties of concrete. J. Build. Mater..

[17] Deng R., Lu X., Ye T. (2024). The effect of JKR surface energy on the flowing property of fresh concrete in DEM simulation. Part. Sci. Technol..

[18] Zhang K., Yu T., Yu W. (2020). Calibration of discrete element parameters for concrete based on JKR contact model. Concrete.

[19] Kleijer A.L., Lasvaux S., Citherlet S., Viviani M. (2017). Product-specific Life Cycle Assessment of ready mix concrete: Comparison between a recycled and an ordinary concrete. Resour. Conserv. Recycl..

[20] Kotwa A. (2015). Effect of selected admixtures on the properties of ordinary concrete. Procedia Eng..

[21] Sarhosis V., Tsavdaridis K.D., Giannopoulos I. (2014). Discrete element modelling (DEM) for masonry infilled steel frames with multiple window openings subjected to lateral load variations. Open Constr. Build. Technol. J..

[22] Deng R. (2016). Research and Application of Rheological Properties of Concrete in Mixer Trucks. Ph.D. Thesis.

[23] Tong Z., Wu H. (2020). Research on wear of conveying pipe of concrete pump based on CFD-DEM coupling. Constr. Mech..

[24] Sun Q., Wang G. (2009). Introduction to Particulate Matter Mechanics.

[25] Yue X.Q., Zhang H., Luo C.S., Shu S., Feng C. (2013). Parallelization of a DEM code based on CPU-GPU heterogeneous architecture. International Conference on Parallel Computing in Fluid Dynamics.

[26] Wei H., Wan H., Yuan S., Liu G., Teng J., Shi N., Liu Z. (2025). A new gelling material: Properties of recycled aggregate concrete under conditions of complete cement replacement using steel slag, ore slag, and fly ash. Constr. Build. Mater..

[27] Khasanov B., Vatin N., Ismailova Z., Mirzaev T. (2020). Physical modification of concrete mix and concrete. IOP Conference Series: Materials Science and Engineering.

[28] Wang Z., Long S., Li Y. (2015). Orthogonal experiment-based parameter group optimization for induction control. J. Mech. Eng..

[29] Cao B. (2025). A Gantry-Type Concrete 3D Printing Additive-Subtractive Manufacturing Device: CN202222491481.3.

[30] Zhao L.J., Feng Z.X., Yao Y., Wang W., Wang J. (2006). Method for determining the installation angle of mixer blades. J. Chang. Univ. (Nat. Sci. Ed.).

[31] Gui Y., Yu Z., Chen H., Gong X., Li W., Che J. (2025). Mold Device for Making Concrete Test Blocks and Construction Method Thereof.

[32] (2021). Test Method for Strength of Cement Mortar (ISO Method).

